# *Sinularia polydactyla* (Ehrenberg, 1834) (Cnidaria, Octocorallia) re-examined, with the description of a new species

**DOI:** 10.3897/zookeys.581.7455

**Published:** 2016-04-14

**Authors:** Leen P. van Ofwegen, Catherine S. McFadden, Yehuda Benayahu

**Affiliations:** 1 Department of Marine Zoology, Naturalis Biodiversity Center, P.O. Box 9517, 2300 RA Leiden, the Netherlands; 2 Department of Biology, Harvey Mudd College, Claremont, CA 91711, USA; 3 Department of Zoology, George S. Wise Faculty of Life Sciences, Tel Aviv University, Ramat Aviv, 69978, Israel

**Keywords:** Alcyonacea, re-description, new species, Indo-Pacific, Red Sea, taxonomy, phylogeny, coral reefs, COI, mtMutS

## Abstract

*Sinularia polydactyla* (Ehrenberg, 1834) is re-described and a lectotype assigned. This led to examination of related material from various Indo-Pacific regions. Consequently, *Sinularia levi* **sp. n.** is described from Eilat, Israel (Gulf of Aqaba, northern Red Sea) and *Sinularia compressa* Tixier-Durivault, 1945 and *Sinularia candidula* Verseveldt and Benayahu, 1983 are synonymized with *Sinularia polydactyla*. Additional specimens identified in the literature as *Sinularia polydactyla* are provisionally reassigned to other taxa.

## Introduction

The Indo-Pacific genus *Sinularia*, with an estimated number of ~190 nominal species, is the most speciose of the zooxanthellate, reef-dwelling octocoral genera (Ofwegen 2002). *Sinularia* species exhibit diverse growth forms and colony sizes, and occupy a wide range of depths from shallow water just below the tideline to deep reef habitats (Fabricius and Alderslade 2001). Occasionally, *Sinularia* colonies form large aggregations, dominating extensive areas on cross-equatorial reefs, including some at the margins of their geographical distribution range (e.g., Benayahu et al. 2012). Some species even deposit large amounts of sclerites in the form of spiculite, and are thus considered to be reef-builders (Jeng et al. 2011). In recent years, along with taxonomic descriptions of new *Sinularia* species, molecular systematic approaches have been applied to resolve species boundaries in the genus (e.g., McFadden et al. 2009). In conjunction with such studies, original type material has also been re-examined, some of which was previously considered lost (e.g., Ofwegen et al. 2013).

During his revision of the soft coral genus *Sinularia*, Verseveldt (1980) could not find the type specimens of several species, and as a result he re-described those species erroneously based on material from specimens of other, but similar-looking species. This became especially obvious with the first molecular phylogenetic study of the genus *Sinularia* (McFadden et al. 2009), in which colonies identified as *Sinularia leptoclados* and *Sinularia polydactyla* showed up in several different clades. As discussed by Ofwegen et al. (2013), *Sinularia leptoclados* (Ehrenberg, 1834) appeared in different sub-clades of Clade 5C presented by McFadden et al. (2009). Likewise, *Sinularia polydactyla* (Ehrenberg, 1834) appeared in several different clades, with specimens from the Red Sea in Clade 4B (characterized by polyps with point sclerites; clubs with central wart distinct, or clubs absent), while Indo-Pacific specimens belonged to Clade 4D (polyps without sclerites; clubs with central wart distinct). Recently we discovered three syntype specimens of *Sinularia polydactyla* in the Zoological Museum of Berlin (ZMB), which were probably overlooked by Verseveldt because they were originally labeled as *Lobularia*. Examination of their sclerites proved two of these syntypes (ZMB 298, 299) to belong to genus *Sinularia* Clade 4D and one of them (ZMB 300) to the genus *Cladiella*. Therefore, we consider the Red Sea specimens previously identified as *Sinularia polydactyla* but belonging to Clade 4B (McFadden et al. 2009) to belong to a yet unknown species, which is described and depicted below. Re-examination of additional material misidentified as *Sinularia polydactyla* revealed another seven specimens belonging to this new species, giving it a distribution from the Red Sea to East Africa (West Indian Ocean).

We also managed to find the material used by Verseveldt (1980) to re-describe *Sinularia polydactyla*, RMNH Coel. 15950. It was also re-examined and found to belong to Clade 4B rather than 4D, and therefore it cannot be *Sinularia polydactyla*.

While examining the syntypes of *Sinularia polydactyla*, it became obvious that *Sinularia compressa* Tixier-Durivault, 1945 and *Sinularia candidula* Verseveldt & Benayahu, 1983 are very similar to *Sinularia polydactyla*. As *Sinularia compressa* was also included in the molecular study and also occurred in two different parts of the phylogenetic tree (McFadden et al. 2009), specimens of these species were also re-examined. Neither these specimens nor the syntypes of *Sinularia compressa* (Verseveldt, 1980: 30) differ much from *Sinularia polydactyla*, and therefore we synonymize *Sinularia compressa* with *Sinularia polydactyla*. The type of *Sinularia candidula* RMNH Coel. 11837, whose original description was accompanied by drawings of sclerites (Verseveldt and Benayahu 1983), was also re-examined in the present study. SEM images of its sclerites are presented below. No distinct differences could be found between specimens of *Sinularia candidula* and specimens previously identified as *Sinularia compressa* or *Sinularia polydactyla* and therefore *Sinularia candidula* is also synonymized with *Sinularia polydactyla*. Other specimens identified as *Sinularia polydactyla* and those with DNA sequences similar to material identified as *Sinularia polydactyla* are also re-examined and discussed.

## Material and methods

### Morphological examination

In order to identify the material, sclerites from different parts of each specimen were obtained by dissolving tissue in 10% sodium hypochlorite, followed by rinsing in fresh water. When appropriate, they were prepared for scanning electron microscopy as follows: the sclerites were carefully rinsed with double-distilled water, dried at room temperature, coated with gold and examined with a Jeol 6480LV electron microscope, operated at 10 kV.

### Abbreviations of museum collections

Material studied is deposited in the Museum für Naturkunde der Humboldt-Universität, Berlin, Germany (ZMB), Naturalis Biodiversity Center (formerly Rijksmuseum van Natuurlijke Historie, Leiden, the Netherlands (RMNH)) and the Zoological Museum, Department of Zoology, Tel Aviv University, Israel (ZMTAU).

### Molecular phylogenetic analyses

Published methods (McFadden et al. 2011) were used to obtain new *mtMutS* and *COI* sequences for specimens ZMTAU Co 36607 and Co 36585, collected in 2014 from Eilat, Israel (Gulf of Aqaba, Red Sea), and material from Guam used by Hoover et al. (2008) (GenBank accession numbers KU230366-KU230389). All other sequences were obtained from GenBank, and have been included in previous phylogenetic analyses (McFadden et al. 2009, 2011, 2014; Haverkort-Yeh et al. 2013) (Suppl. material 2). Sequences were aligned using the L-INS-i method in MAFFT (Katoh et al. 2005), and evolution models were selected for each gene separately using jModeltest (Guindon and Gascuel 2003, Darriba et al. 2012). Maximum likelihood analyses were run using Garli 2.0 (Zwickl 2006) for *mtMutS* alone and in a combined analysis of *mtMutS* plus *COI* with different models of evolution applied to each data partition (*mtMutS*: HKY+G; *COI*: HKY+I). Bayesian analyses of the separate (*mtMutS*) and combined (*mtMutS* + *COI*) data sets were run using MrBayes v. 3.2.1 (Ronquist et al. 2012) with the same evolution models applied to separate data partitions. Bayesian analyses were run for 2 million generations (until standard deviation of split partitions < 0.01) with a burn-in of 25% and default Metropolis coupling parameters. MEGA v.5 (Tamura et al. 2011) was used to calculate pairwise measures of genetic distance (Kimura 2-parameter) among sequences.

### Molecular phylogenetic results

**Figure 1.**
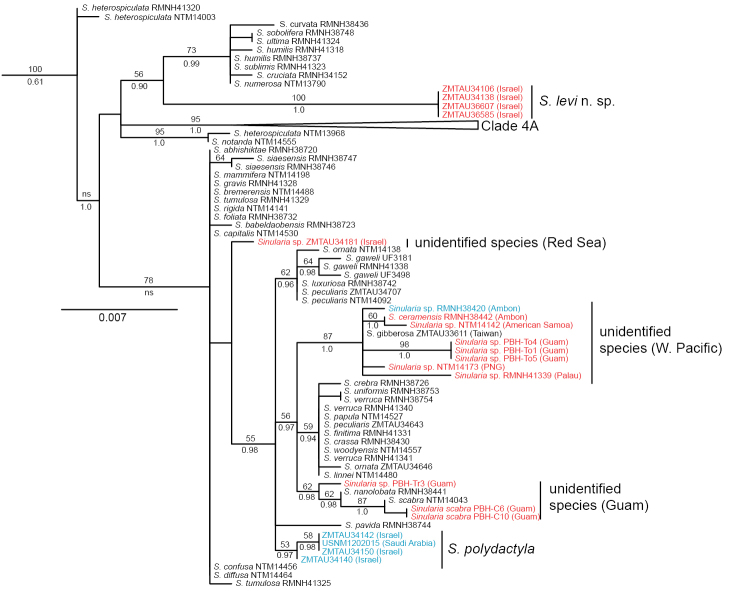
Maximum likelihood tree of *Sinularia* Clade 4 (McFadden et al. 2009) based on 735 bp of *mtMutS* sequence. Outgroup (*Sinularia* Clade 2) not shown. Numbers above branches are ML bootstrap percentages; numbers below branches are posterior probabilities from Bayesian Inference. Red: specimens identified in previous work as *Sinularia polydactyla*; blue: specimens identified in previous work as *Sinularia compressa*.

Phylogenetic analyses of *mtMutS* included sequences for 76 specimens identified as 44 morphospecies belonging to *Sinularia* Clade 4 (McFadden et al. 2009); an additional six specimens representing four morphospecies belonging to Clade 2 served as the outgroup. A total of 20 specimens had previously been identified as either *Sinularia polydactyla* or *Sinularia compressa*. Those specimens fell into five separate clades within the *mtMutS* gene tree (Figure 1). Four specimens from Eilat, Israel, previously identified as *Sinularia polydactyla*, belonged to a well-supported, genetically distinct sub-clade within Clade 4B. Mean genetic distances (Kimura 2-parameter) between this clade and other clades containing specimens of *Sinularia polydactyla* or *Sinularia compressa* ranged 3.0–3.8% (Table 1). Four additional specimens from the Red Sea, all previously identified as *Sinularia compressa*, belonged to a moderately well-supported clade within Clade 4D. Mean genetic distances between this clade and others ranged 0.7–3.0%. Another nine specimens, all from the western Pacific, belonged to a different well-supported clade within Clade 4D. These included seven specimens identified previously as *Sinularia polydactyla*, one *Sinularia compressa*, and a specimen of *Sinularia gibberosa*. Mean genetic distances between this clade and other clades of *Sinularia polydactyla* and *Sinularia compressa* ranged 1.1–3.8%. Three specimens from Guam, however, fell outside of this western Pacific clade and grouped instead with specimens identified as *Sinularia scabra* and *Sinularia nanolobata*. Finally, a single specimen of *Sinularia polydactyla* from Eilat, Israel (ZMTAU Co 34181) belonged to none of these clades, differing from these by >0.7%. The phylogenetic position of ZMTAU Co 34181 within the *mtMutS* tree was poorly resolved, but genetically it was closer to species in Clade 4C than to those in Clade 4D.

**Table 1.** Mean genetic distances (Kimura 2-parameter, ± s.d.) among *mtMutS* sequences within and between the clades of *Sinularia* highlighted in Fig. 1.

	*Sinularia levi* sp. n.	*Sinularia polydactyla*	W. Pacific clade	Guam clade
***Sinularia levi* sp. n.**	0.000 ± 0.0000			
***Sinularia polydactyla***	0.030 ± 0.0006	0.001 ± 0.0007		
**W. Pacific clade**	0.038 ± 0.0026	0.011 ± 0.0023	0.005 ± 0.0031	
**Guam clade**	0.035 ± 0.0007	0.009 ± 0.0021	0.013 ± 0.0028	0.005 ± 0.0040
**ZMTAU 34181**	0.031 ± 0.0000	0.007 ± 0.0007	0.013 ± 0.0023	0.011 ± 0.0024

*COI* sequences were available for only 42 of the 76 Clade 4 specimens, representing 24 morphospecies and 15 of 20 individuals of *Sinularia polydactyla* and *Sinularia compressa*. Results of the combined analysis of *mtMutS* and *COI* for this more limited dataset were congruent with and provided stronger ML bootstrap support for the same clades of *Sinularia polydactyla* / *Sinularia compressa* identified in the *mtMutS* tree (Suppl. material 1).

Genetic distances among specimens in each of the two Red Sea clades ranged from 0–0.1%, suggesting that each of those clades represents a single species (Table 1). Within the clade of western Pacific specimens, however, genetic distances ranged 0–1.1%. Intraspecific variation in *mtMutS* is rarely >0.5% (McFadden et al. 2011, 2014), which suggests that this clade may comprise more than one species. RMNH Coel. 41339 from Palau differed from all other specimens in the western Pacific clade by ≥0.5%, as also did three specimens from Guam. Among the three specimens from Guam that did not belong to the western Pacific clade, one (PBH-Tr3) differed from the other two by 0.7%, suggesting that it represents yet another different species.

## Taxonomy

### Sinularia polydactyla

Taxon classificationAnimaliaAlcyonaceaAlcyoniidae

(Ehrenberg, 1834)

Figures 2A–C, F–G , 3 , 4 , 5 , 6 , 7 , 8 , 9 , 10 , 11 , 12 , 13 , 14

Lobularia polydactyla Ehrenberg, 1834: 58 (Red Sea).
? Sinularia polydactyla ; Benayahu and Schleyer 1996: 6 (Mozambique); Benayahu et al. 2003: 56 (Mozambique); Dautova and Savinkin 2013: 220 (Vietnam).
Sinularia polydactyla (partly); Benayahu et al. 2002: 278 (Red Sea).
Sinularia compressa Tixier-Durivault, 1945: 150 (Red Sea); Verseveldt 1980: 30 (older literature); Benayahu et al. 2002: 278; 2003: 55 (Mozambique); Samimi Namin and Ofwegen 2009: 8 (Persian Gulf); Haverkort-Yeh et al. 2013: 286 (Red Sea).
Sinularia compressa (partly); McFadden et al. 2009: 318; 2011: 25; Benayahu et al. 2013: 1544.
Sinularia candidula Verseveldt & Benayahu, 1983: 11 (Red Sea).
Sinularia polydactyla NOT Sinularia polydactyla; Verseveldt 1971: 4 (Madagascar); Tixier-Durivault 1972: 677 (Reunion; = Sinularia shlagmani Benayahu & Ofwegen, 2012); Verseveldt 1972: 457 (Eniwetok Atoll, Marshall Islands); 1974: 96 (New Caledonia): 1977: 3 (Fiji, Guam, Samoa); 1978: 50 (Guam, Palau); 1980: 108 (older literature); Ofwegen and Benayahu 1992: 140 (Tanzania); Ofwegen and Vennam 1994: 138 (Ambon); Benayahu 1995: 107 (Ryukyu Archipelago); Ofwegen 1996: 208 (Bismarck Sea); Benayahu 1997: 237 (Guam, *in situ* image); 2002: 20 (Ryukyu Archipelago); Benayahu et al. 2004: 551 (Taiwan; *in situ* image); Manuputty and Ofwegen 2007: 192 (Ambon; = Sinularia ceramensis); McFadden et al. 2009: 321; 2011: 25; Benayahu and Ofwegen 2011: 118 (Singapore); Benayahu et al. 2013: 1544.
Sinularia polydactyla NOT Sinularia compressa; Benayahu 1997: 215 (Guam); 2002: 18 (Japan); Benayahu et al. 2004: 551 (Taiwan); Manuputty and Ofwegen 2007: 191 (Ambon); Benayahu and Chou 2010: 4 (Singapore).

#### Type material examined.

ZMB 299, lectotype (herein designated), Red Sea, leg. Hemprich, Ehrenberg; ZMB 298, two paralectotypes, same data as holotype; ZMB 300, same data as holotype.

#### Other material examined.

RMNH Coel. 8890, Gulf of Aqaba, Red Sea, 1.5 km N of Saudi Arabian border, 50–70 cm, 10–20 m from coast, 15 February 1972, coll. H. Schumacher, det. J. Verseveldt, one specimen and two microscope slides; RMNH Coel. 8891, Gulf of Aqaba, Red Sea, 1.5 km N of Saudi Arabian border, 80 cm, 18 February 1972, coll. H. Schumacher, det. J. Verseveldt, one specimen and two microscope slides; RMNH Coel. 8892, Marsa el Muqeibla (= Makbala), Gulf of Aqaba, Red Sea, from reef wall, 6 January 1968, coll. Hebrew University, Jerusalem - Smithsonian Red Sea project 63/SLR 1147, det. J. Verseveldt, one specimen and 3 microscope slides; RMNH Coel. 8944, Marsa abu Zabad, Gulf of Aqaba, Red Sea, 15 September 1967, coll. Hebrew University, Jerusalem - Smithsonian Red Sea project, det. J. Verseveldt, one specimen and five microscope slides; RMNH Coel. 8951, Marsa el Maqeilba, Gulf of Aqaba, Red Sea, 6 January 1968, coll. Hebrew University, Jerusalem - Smithsonian Red Sea project, det. J. Verseveldt, one specimen and four microscope slides; ZMTAU Co 25287, Red Sea, Gulf of Aqaba, Nakeb Shahin, 25 m, coll. Y. Benayahu, 29 November 1981; ZMTAU Co 25309, Red Sea, southern tip of Sinai Peninsula, Sharm El Sheikh, 25 m, coll. Y. Benayahu, 30 November 1981; ZMTAU Co 25378, Red Sea, Gulf of Aqaba, Nakeb Shahin, 18-24 m, coll. Y. Benayahu, 5 November 1981; ZMTAU Co 25419, Red Sea, Gulf of Aqaba, Taba, 1 m, coll. Y. Benayahu, 30 July 1984; ZMTAU Co 26119, Red Sea, North, Tawila Island, 6 m, coll. Y. Benayahu, 24 September 1989; ZMTAU Co 31609, Red Sea, Eritrea, Dahlak Archipelago, Dahlak Island, channel in front of Lul hotel, coll. M. Schleyer, 12 February 1998; ZMTAU Co 31610, Red Sea, Eritrea Dahlak Archipelago, Intere Island, 15°38.504'N, 39°53.580'E, 12.5 m, coll. M. Schleyer, 3 May 1997; ZMTAU Co 32947, Red Sea, Eritrea, Dahlak Archipelago, between Nocra Island and Dahlak Island, southern entrance to the channel, 15°41.60'N, 39°56.40'E, 2–3 m, coll. Y. Benayahu, 15 February 2005; ZMTAU Co 32961, Red Sea, Eritrea, Dahlak Archipelago, Shumma Island, 15°32.00'N, 40°00.00'E, 8–12 m, coll. Y. Benayahu, 16 February 2005; ZMTAU Co 33104, Israel, Gulf of Aqaba, Eilat, Marine laboratory, reef off the Inter University Institute for Marine Sciences, 50 m, coll. S. Einbinder, 8 June 2004; ZMTAU Co 35301, Israel, Gulf of Aqaba, Eilat, reef off the Inter University Institute for Marine Sciences, 14 m, coll. Y. Benayahu, 19 January 2011; *Sinularia compressa* material: ZMTAU 34140, ZMTAU 34142, and ZMTAU 34150 used by McFadden et al. (2011).

#### Re-description.

**Figure 2.**
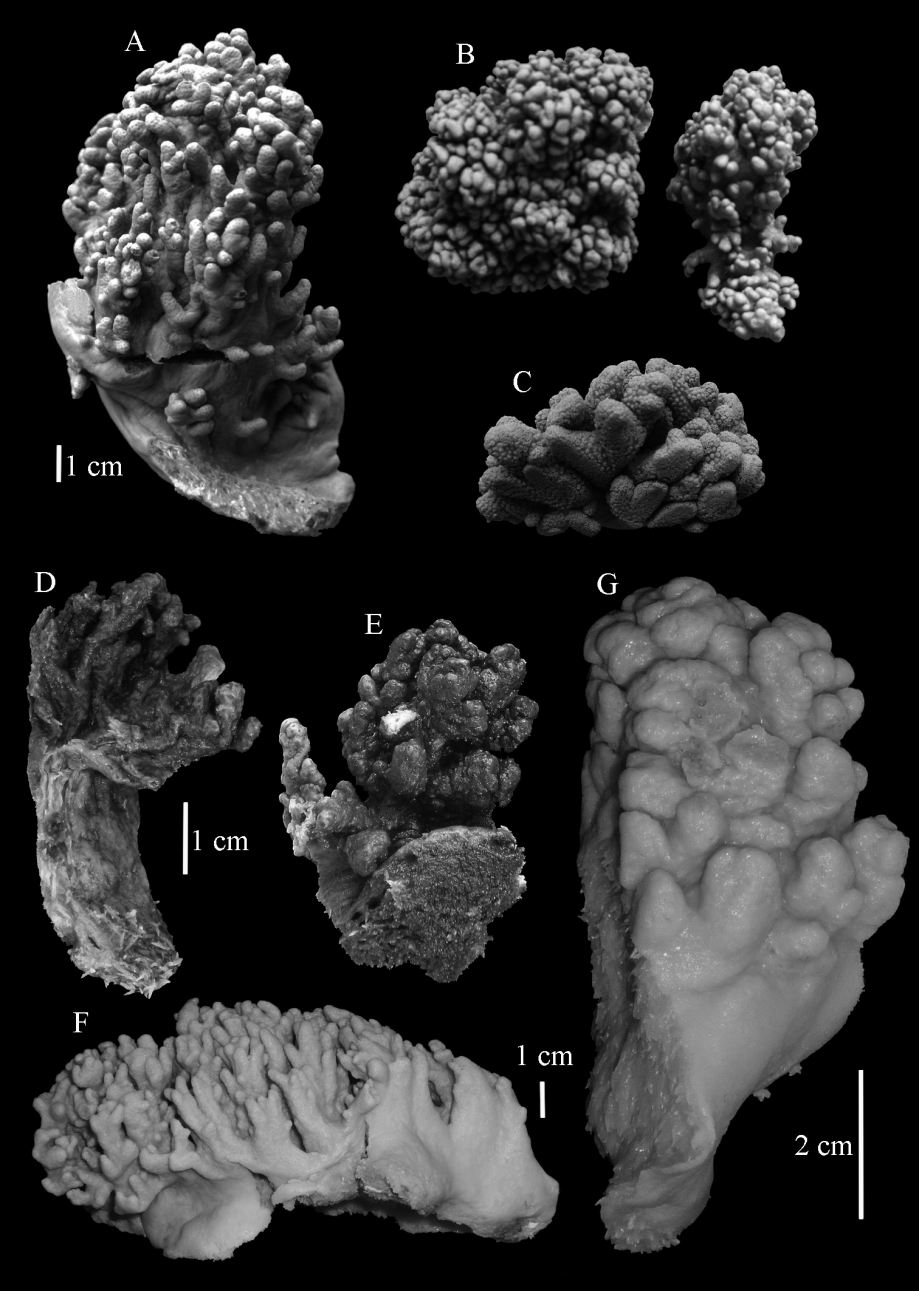
Colonies. **A** *Sinularia polydactyla* (Ehrenberg, 1834), lectotype ZMB 299 **B** paralectotype ZMB 298 **C** paralectotype ZMB 300 **D** *Sinularia levi* sp. n. holotype ZMTAU Co 34106 **E** paratype ZMTAU Co 34138 **F** *Sinularia compressa* Tixier-Durivault, 1945, ZMTAU 31610 **G** ZMTAU 34142.

The lectotype is 14.5 cm high and 9 cm wide (Figure 2A). The primary lobes give off short finger-like lobules up to 1 cm long. The polyp openings are visible as small pits.

**Figure 3.**
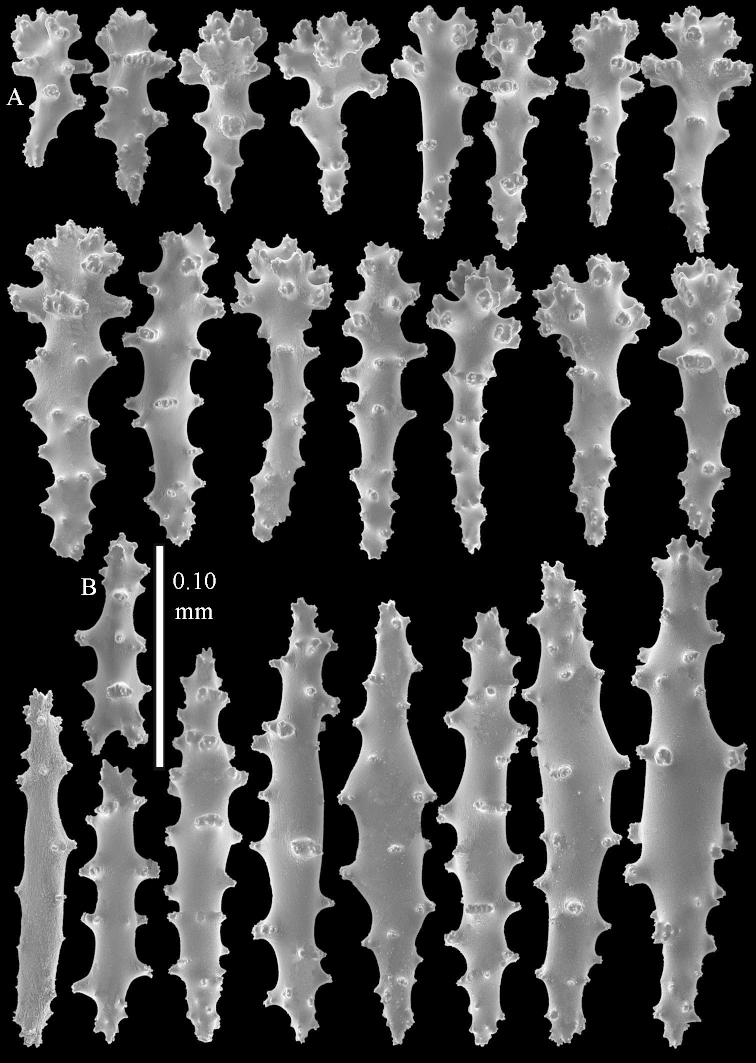
*Sinularia polydactyla* (Ehrenberg, 1834), lectotype ZMB 299. **A** clubs of surface layer top of colony **B** spindles.

**Figure 4.**
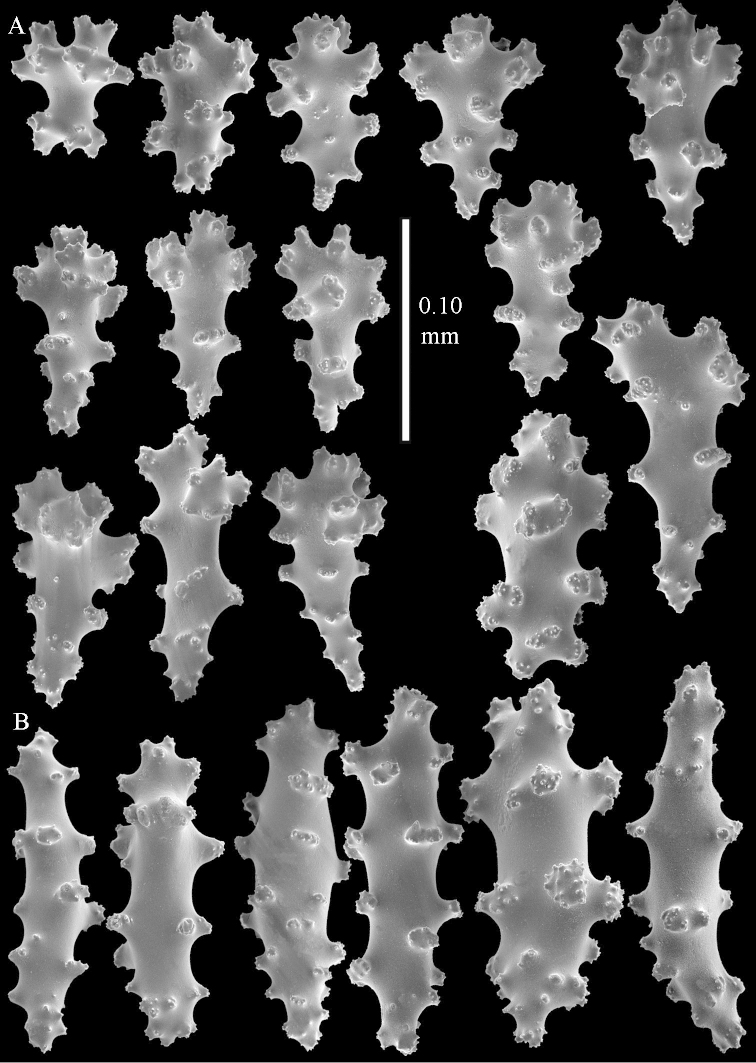
*Sinularia polydactyla* (Ehrenberg, 1834), lectotype ZMB 299. **A** clubs of surface layer base of colony **B** spindles.

**Figure 5.**
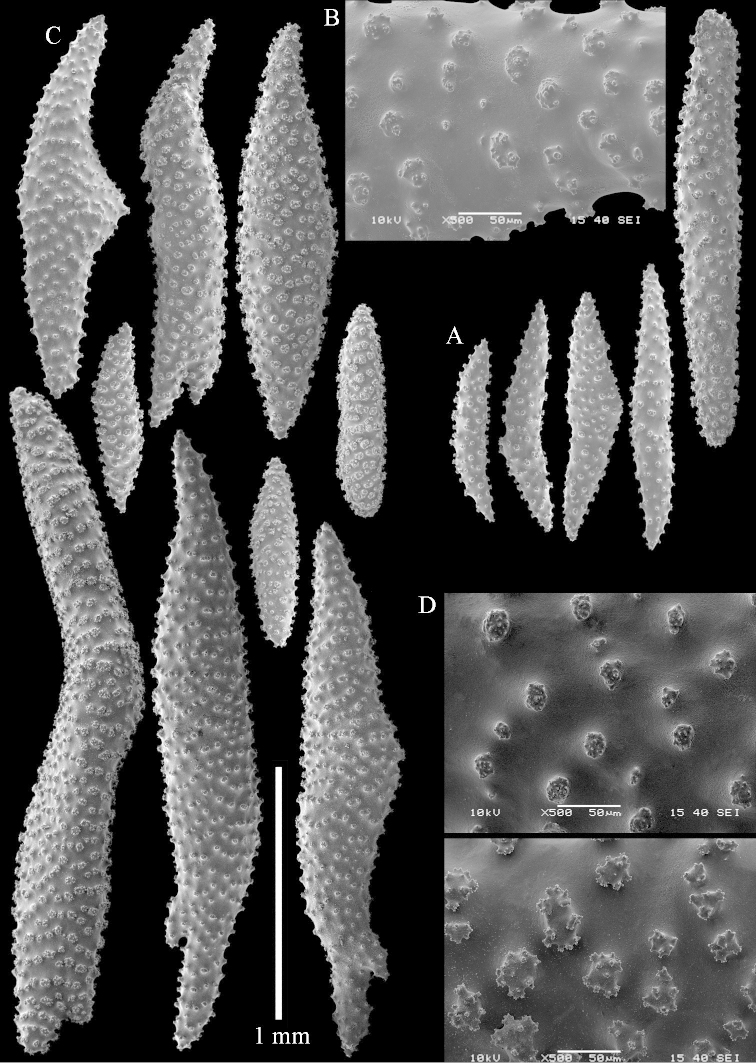
*Sinularia polydactyla* (Ehrenberg, 1834), lectotype ZMB 299. **A** spindles of interior of top of colony **B** tuberculation of one of the spindles **C** spindles of the interior of base of the colony **D** tuberculation of the spindles.

**Sclerites.** Polyps without sclerites. The surface layer of the lobules has clubs with a distinct central wart, the smallest are 0.07 mm long, most are around 0.10 mm, but some reach even a length of 0.15 mm (Figure 3A). Furthermore, the surface layer of the lobules has spindles, up to 0.25 mm long, with simple tubercles (Figure 3B). The sclerites of the surface layer of the base of the colony resemble those of the surface layer of the lobules, but the clubs are much shorter, only up to 0.10 mm long, with wider handles. The spindles are also wider and shorter than those of the top of the colony, up to 0.15 mm long (Figure 4). The interior of the colony has unbranched spindles. In the lobules the spindles are up to 2 mm long (Figure 5A), featuring simple or complex tubercles (Figure 5B). In the base of the colony they are up to 3 mm long (Figure 5C), many with more complex tubercles (Figure 5D).

**Colour.** The alcohol-preserved specimen is light brown.

#### Remarks.

**Figure 6.**
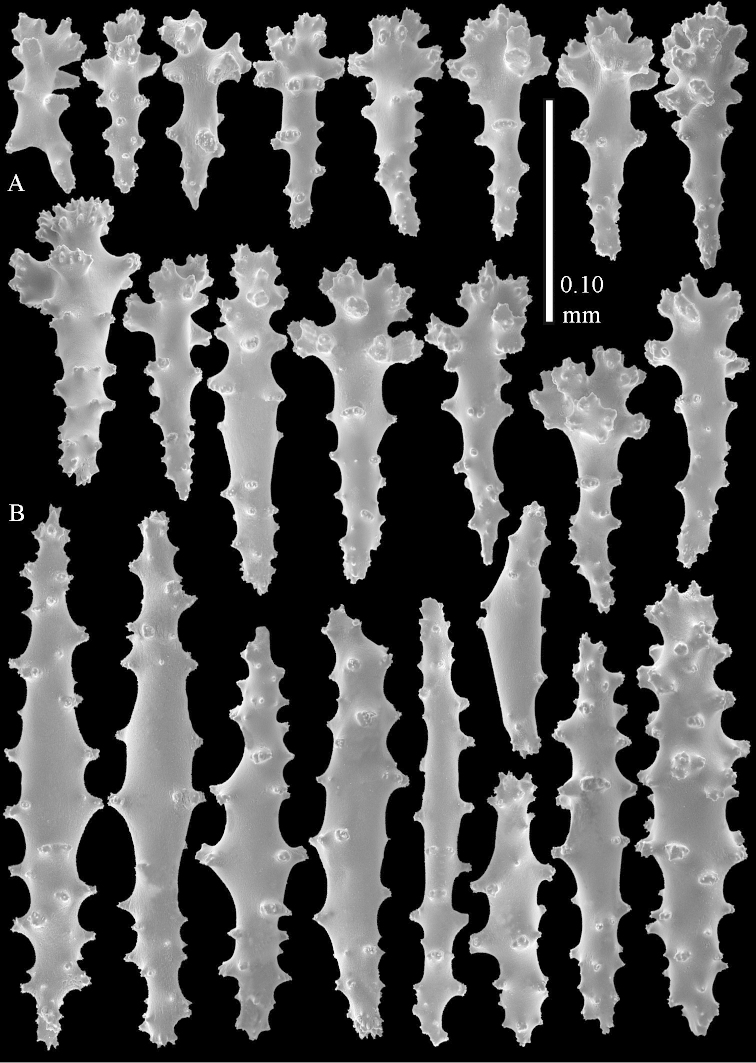
*Sinularia polydactyla* (Ehrenberg, 1834), paralectotype ZMB 298 (smallest colony). **A** clubs of surface layer top of colony **B** spindles.

**Figure 7.**
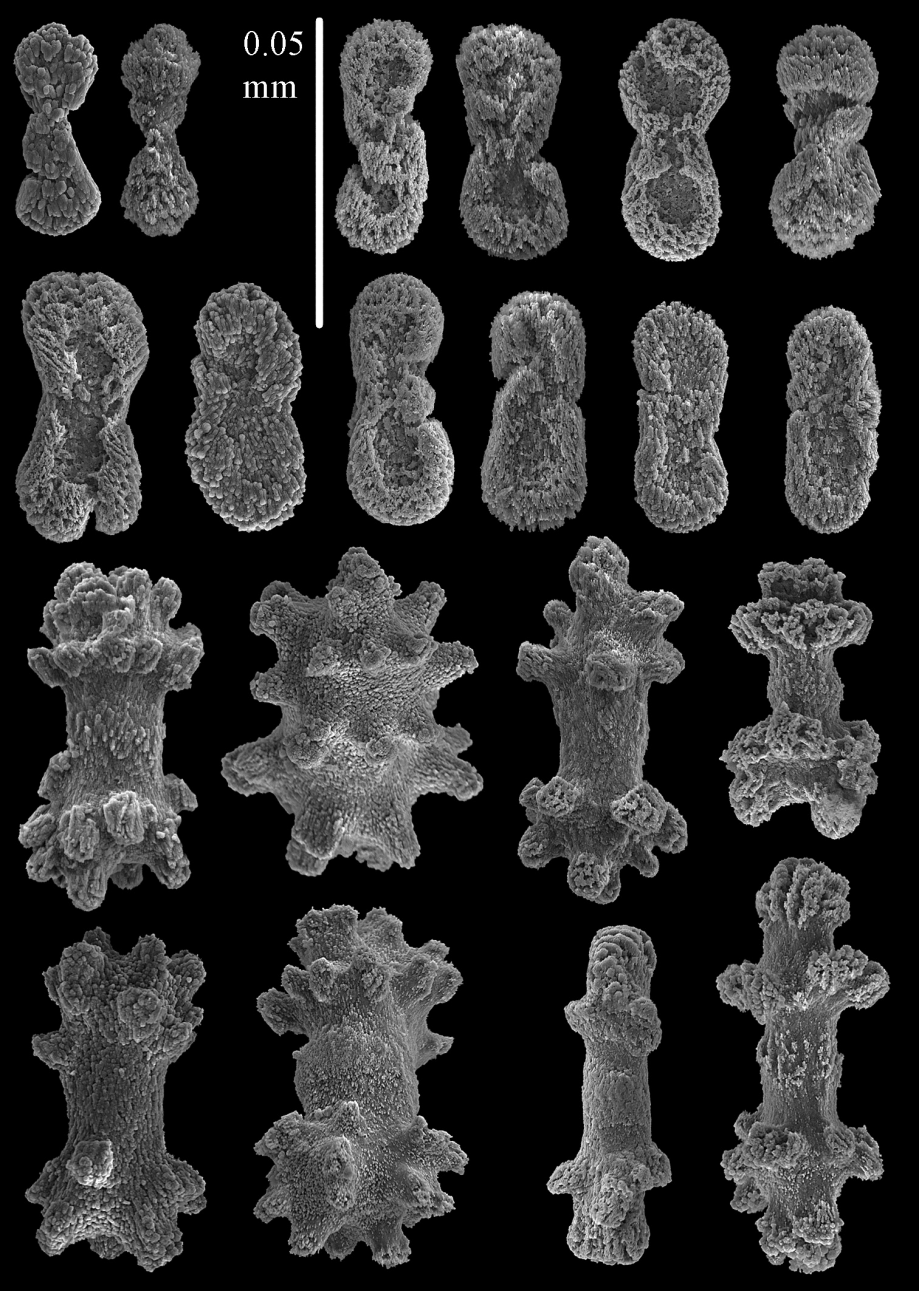
*Sinularia polydactyla* (Ehrenberg, 1834), paralectotype ZMB 300. Sclerites of top of colony.

**Figure 8.**
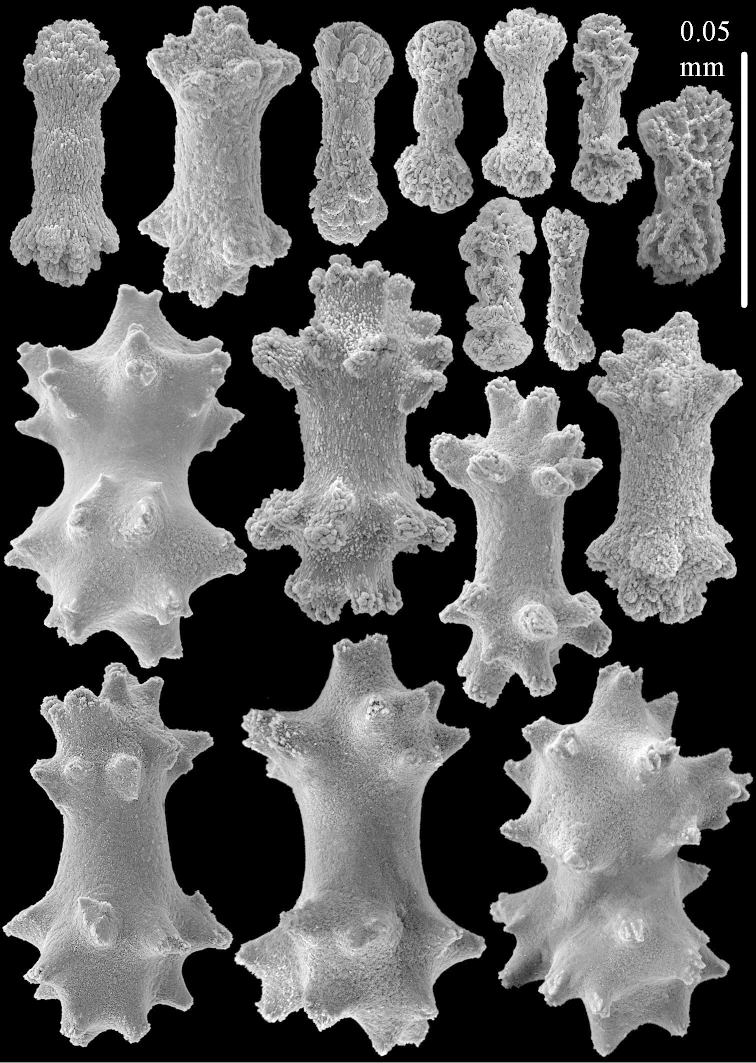
*Sinularia polydactyla* (Ehrenberg, 1834), paralectotype ZMB 300. Sclerites of base of colony.

The two paralectotypes ZMB 298 are smaller than the lectotype (Figure 2B) but the sclerites are similar (Figure 6). Paralectotype ZMB 300 is not a *Sinularia*, but a *Cladiella* specimen, as proven by its colony shape and typical suite of figure-eight and dumbbell sclerites (Figures 2C, 7–8).

Notably the Red Sea *Sinularia polydactyla* colonies can be much larger than the lectotypes and have longer lobules (Figure 2F, ZMTAU 31610).

**Figure 9.**
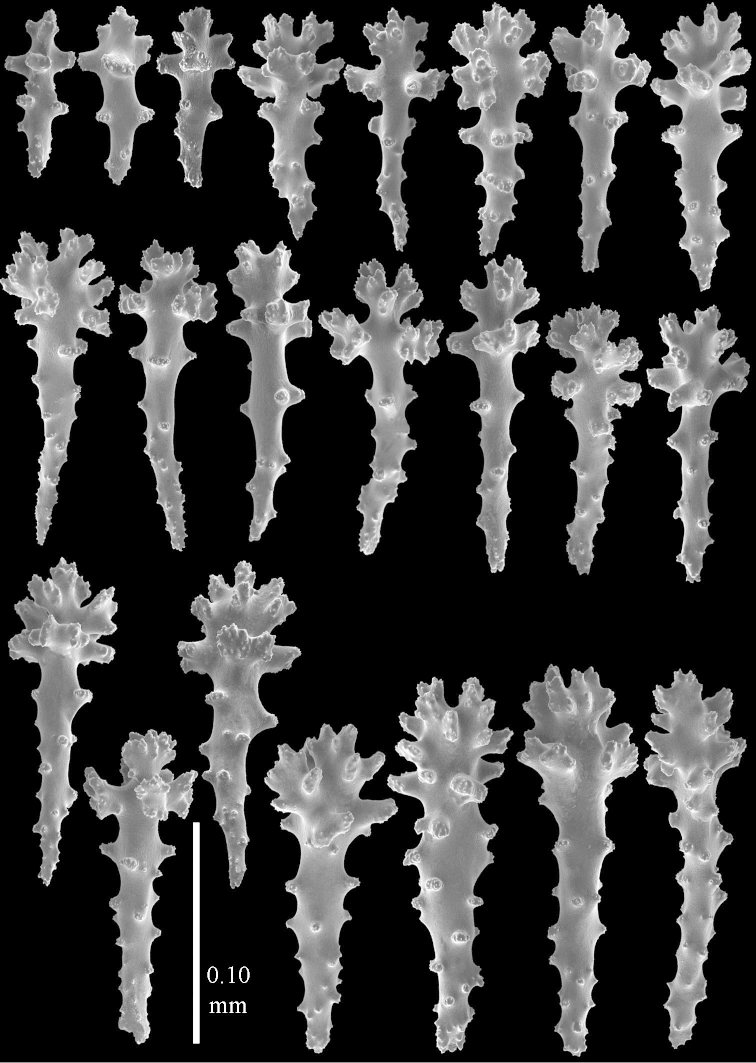
*Sinularia compressa* Tixier-Durivault, 1945, ZMTAU 34142. Clubs of surface layer of top of colony.

**Figure 10.**
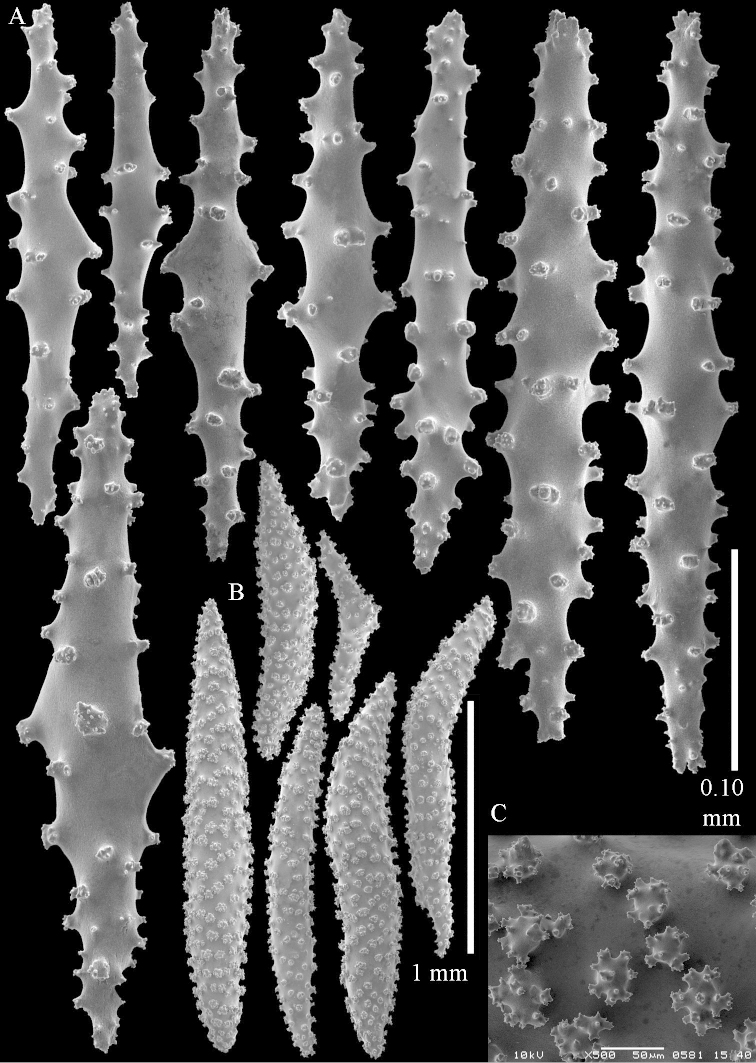
*Sinularia compressa* Tixier-Durivault, 1945, ZMTAU 34142. **A** spindles of surface layer of top of colony **B** spindles of interior of top of colony **C** tuberculation of a spindle.

**Figure 11.**
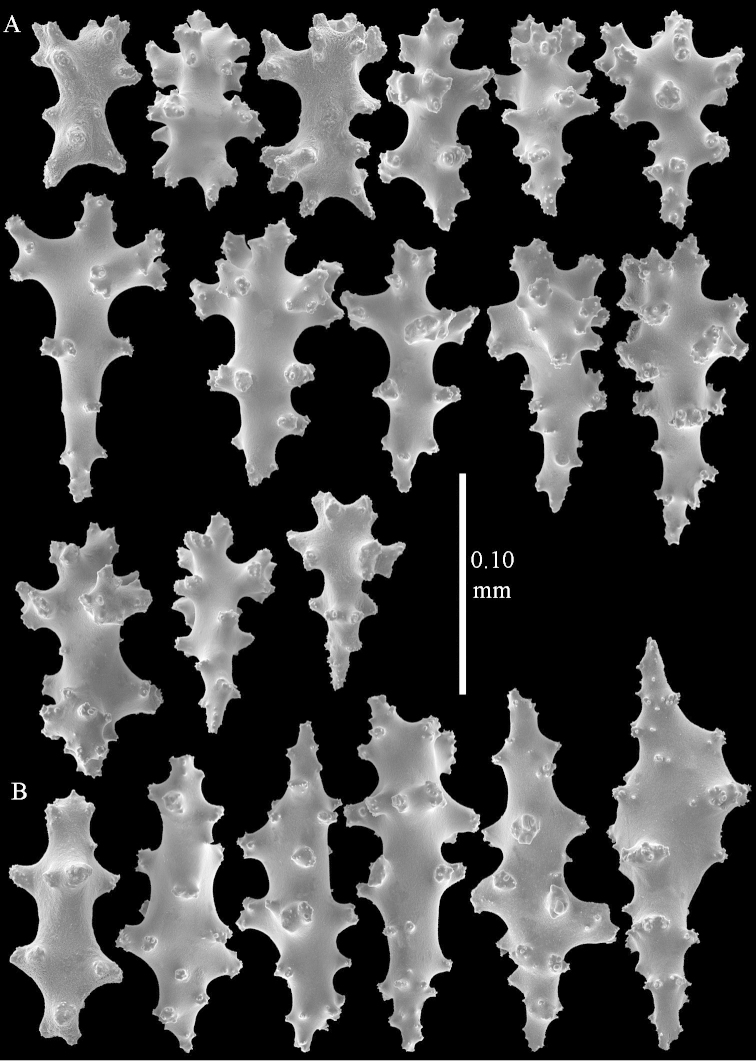
*Sinularia compressa* Tixier-Durivault, 1945, ZMTAU 34142. **A** clubs of surface layer base of colony **B** spindles.

*Sinularia compressa* Tixier-Durivault, 1945 exhibits close similarity to *Sinularia polydactyla*. It differs in having clubs in the surface layer of the lobes with more slender handle and spinier head. *Sinularia compressa* specimens ZMTAU 34140, 34142, and 34150, all from the Red Sea (Figure 2G) feature similar sclerites (Figures 9–11) despite differences in their colony shape.

**Figure 12:**
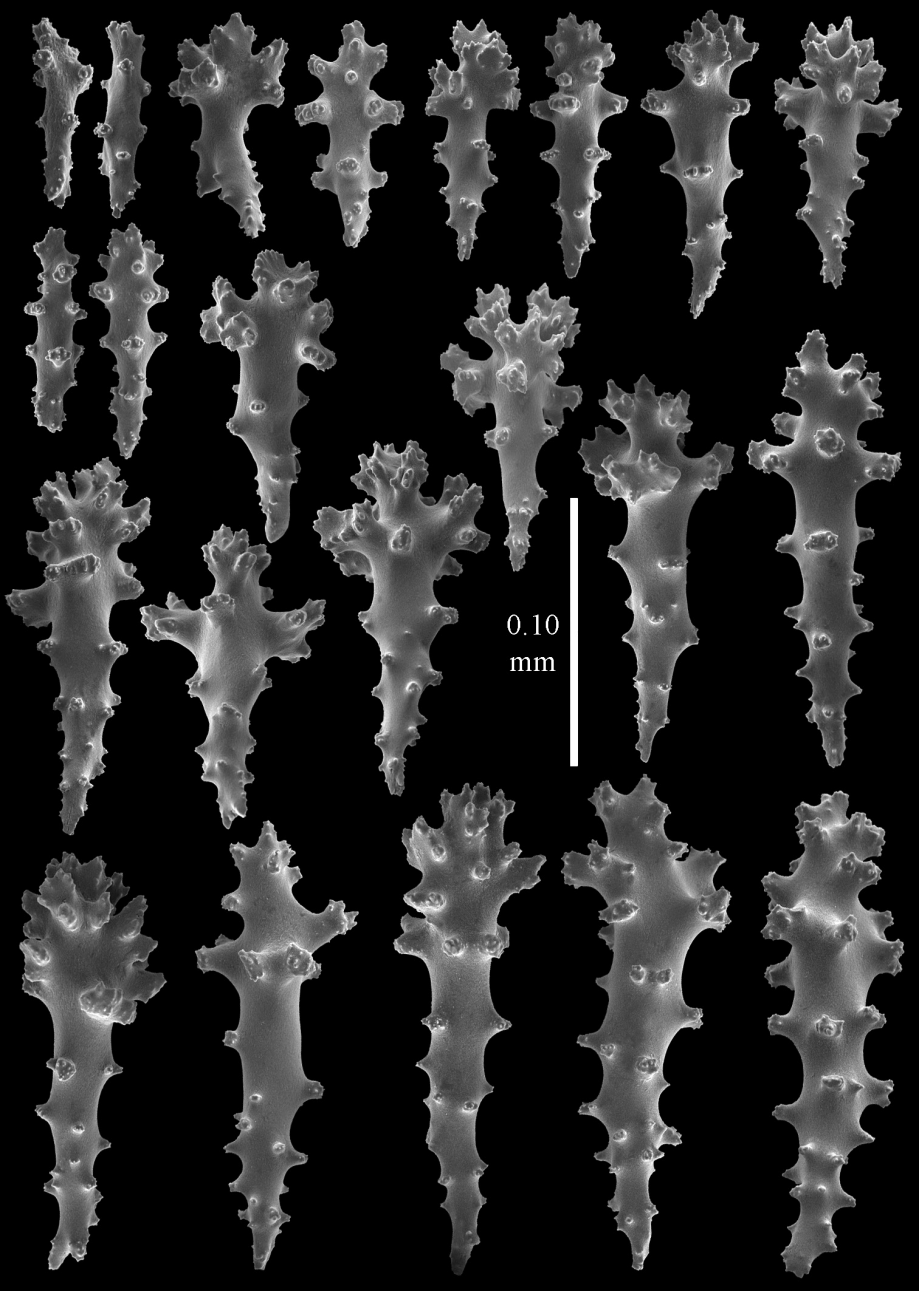
*Sinularia candidula* Verseveldt & Benayahu, 1983. Clubs of surface layer top of colony.

**Figure 13:**
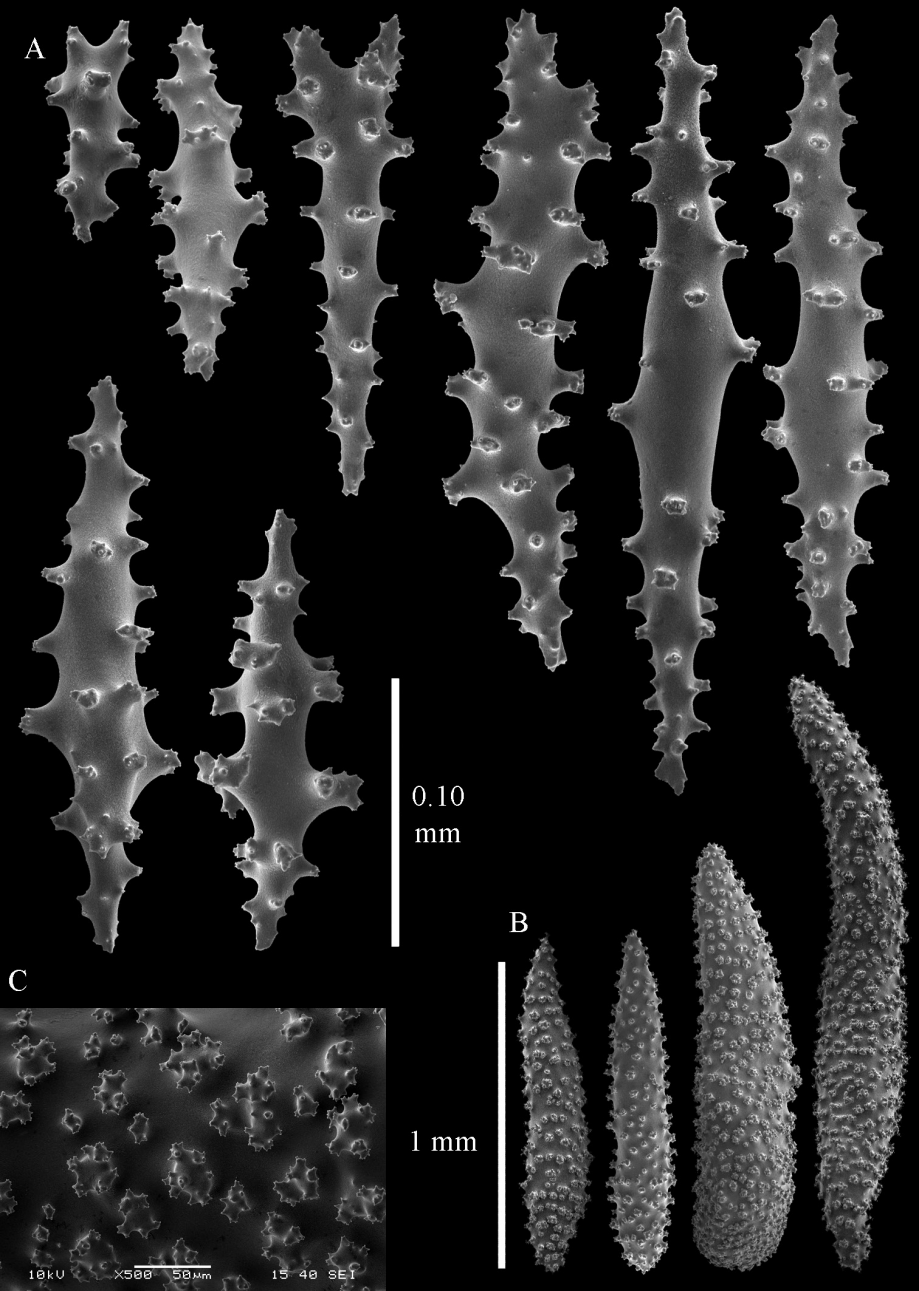
*Sinularia candidula* Verseveldt & Benayahu, 1983. **A** spindles of surface layer of top of colony **B** spindles of interior of top of colony **C** tuberculation of a spindle.

**Figure 14:**
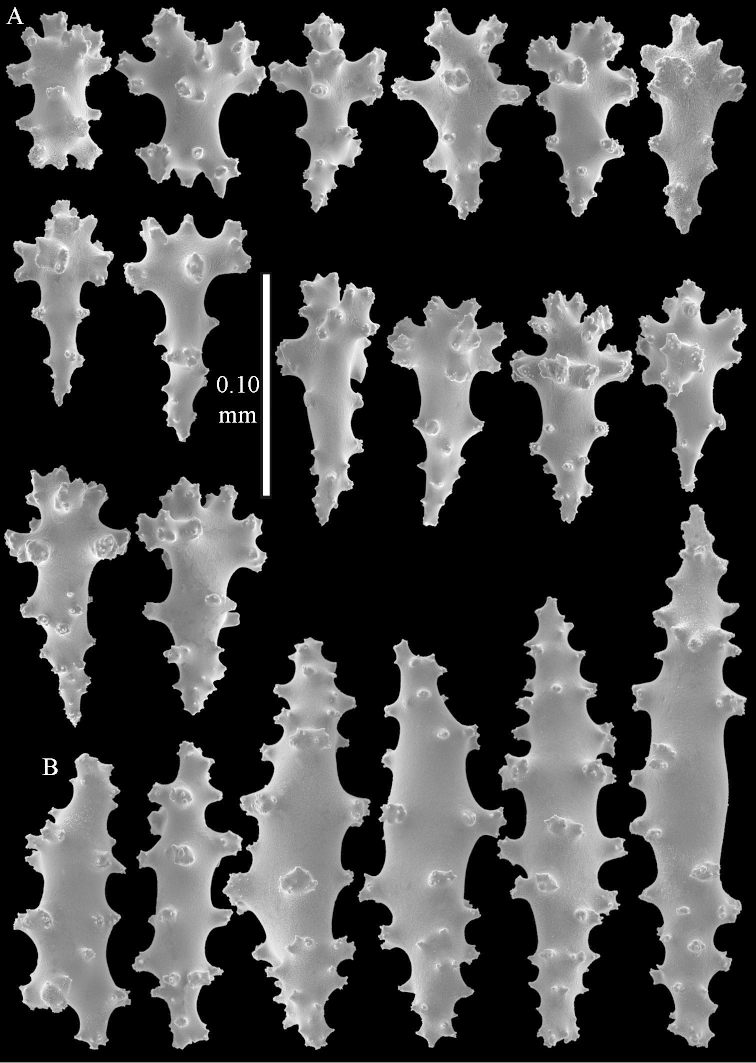
*Sinularia candidula* Verseveldt & Benayahu, 1983. **A** clubs of surface layer base of colony **B** spindles.

Finally, we re-examined the type of *Sinularia candidula* Verseveldt & Benayahu, 1983, RMNH Coel. 11837, also depicting its sclerites (Figures 12–14). There were no noticeable differences between that species and specimens identified as *Sinularia polydactyla*, and therefore we synonymized *Sinularia candidula* also with *Sinularia polydactyla*.

### Sinularia levi sp. n.

Taxon classificationAnimaliaAlcyonaceaAlcyoniidae

http://zoobank.org/1EBC5A7A-629C-4A43-8A1D-C5A45B1B494B

**Figure 15.**
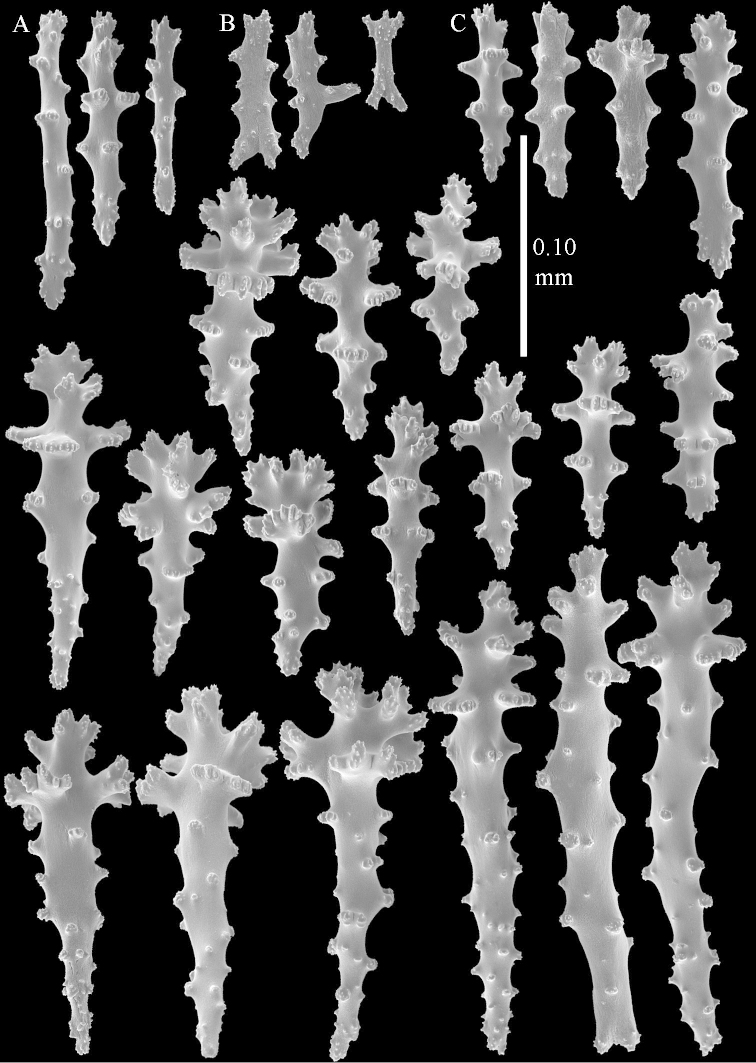
*Sinularia levi* sp. n. holotype, ZMTAU Co 34106. **A** point clubs **B** tentacle rods **C** clubs of surface layer top of colony.

**Figure 16.**
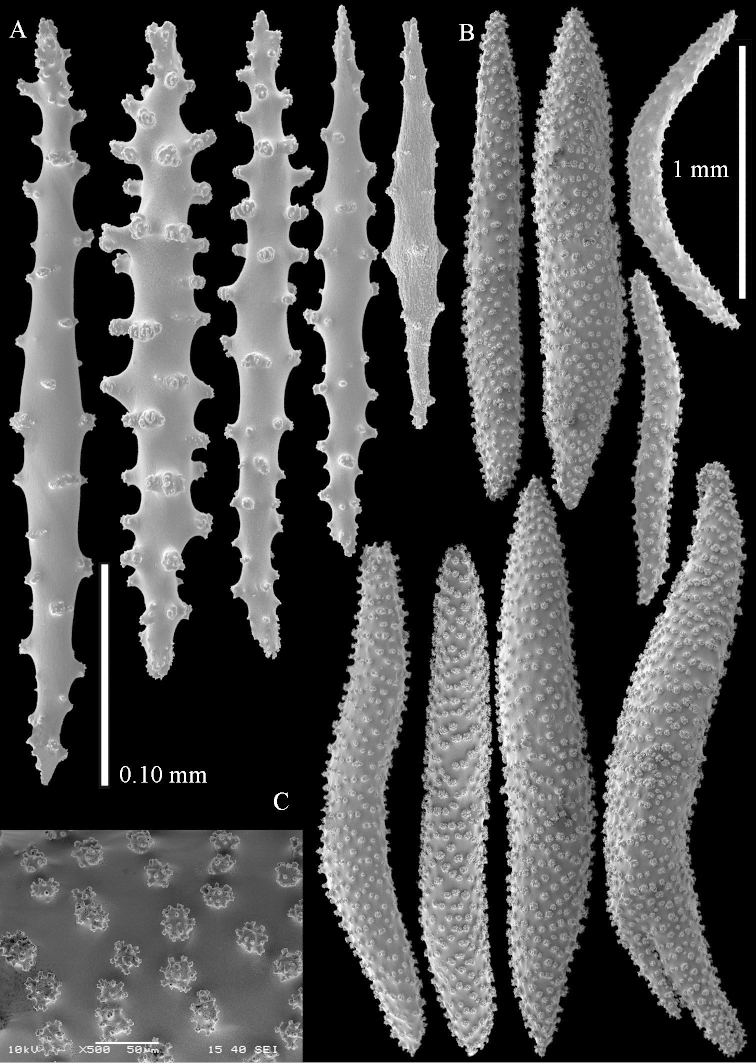
*Sinularia levi* sp. n. holotype, ZMTAU Co 34106. **A** spindles of surface layer top of colony **B** spindles of the interior of top of colony **C** tuberculation of a spindle.

**Figure 17.**
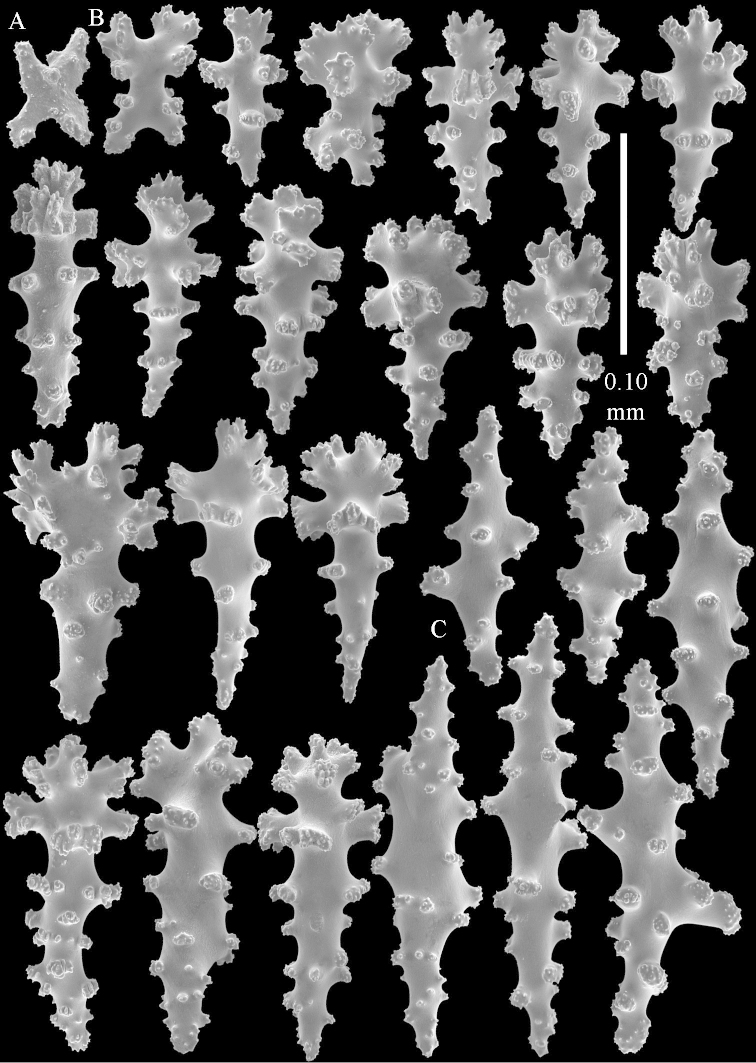
*Sinularia levi* sp. n. holotype, ZMTAU Co 34106. **A** cross of surface layer of the base of the colony **B** clubs **C** spindles.

**Figure 18.**
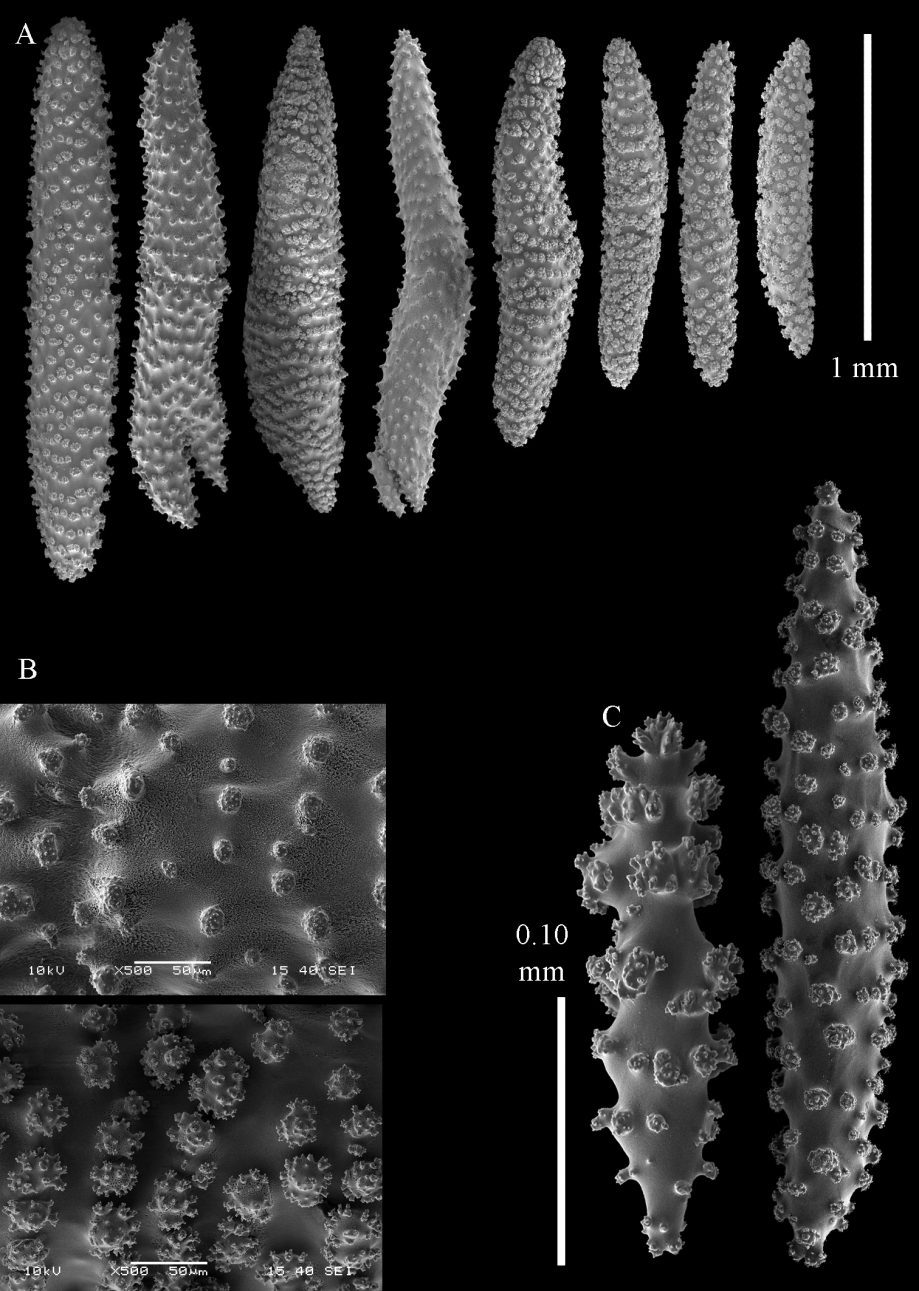
*Sinularia levi* sp. n. holotype, ZMTAU Co 34106. **A** spindles of interior of base of colony **B** tuberculation of two of the spindles **C** spindle and club intermediate between surface and interior sclerites.

Figures 2D–E , 15 , 16 , 17 , 18 , 44

Sinularia polydactyla (partly); Verseveldt 1971: 4 (Madagascar).
Sinularia polydactyla ; McFadden et al. 2009: 321 (Eilat, northern Red Sea); 2011: 25.

#### Type material examined.

holotype: ZMTAU Co 34106, Eilat Nature Reserve, Gulf of Aqaba, northern Red Sea (Israel), 29°30.6'N, 34°55.35'E, depth 2.4-5.5 m, coll. Y. Benayahu, 24 July 2007; paratype: ZMTAU Co 34138, same data as holotype.

#### Other material examined.

RMNH Coel. 6648, W of harbour, Hellville, Nosy Bé, Madagascar, 12 m, 26 July 1967, coll. A.G. Humes, 1205, det. J. Verseveldt, one specimen and six microscope slides; RMNH Coel. 6649, Ambariobe, near Nosy Bé, Madagascar, 2 m, 22 August 1967, coll. A.G. Humes, 1307, det. J. Verseveldt, one specimen and four microscope slides; RMNH Coel. 6650, Banc de Cinq Mètres, near Nosy Bé, Madagascar, 20 m, 6 August 1967, coll. A.G. Humes, det. J. Verseveldt, one specimen and four microscope slides; RMNH Coel. 6651, Banc de Cinq Mètres, near Nosy Bé, Madagascar, 20 m, 6 August 1967, coll. A.G. Humes, det. J. Verseveldt, one specimen and three microscope slides; ZMTAU 34108, Eilat, Gulf of Aqaba, northern Red Sea, Israel, 29°30.6'N, 34°55.35'E, 2.4–5.5 m, 24 July 2007, coll. Y Benayahu; ZMTAU 36585, Eilat, Gulf of Aqaba, northern Red Sea, Israel,1–2 m, June 2014, coll. E. Shoham and Y. Benayahu; ZMTAU 36607, Eilat, Gulf of Aqaba, northern Red Sea, Israel, 1–2 m, June 2014, coll. E. Shoham and Y. Benayahu.

#### Description.

The holotype is 5.5 cm high and 3 cm wide (Figure 2D) with a stalk 3 cm long. The primary lobes give off short knob-like lobules up to 5 mm long. The polyp openings are visible as small pits.

**Sclerites.** Polyps without collaret, but with points featuring poorly developed clubs, up to 0.15 mm long (Figure 15A). Tentacles with rods that sometimes are ramified, up to 0.08 mm long (Figure 15B). The surface layer of the lobules has clubs with a central wart, the smallest are 0.08 mm long, most are around 0.10 mm, some reach a length of 0.25 mm (Figure 15C). Furthermore, the surface layer of the lobules has spindles, up to 0.35 mm long, with simple tubercles (Figure 16A). The sclerites of the surface layer of the base of the colony resemble those of the surface layer of the lobules, but clubs and spindles are shorter, up to 0.20 mm long, and the spindles and handles of the clubs are wider (Figure 17). A few sclerites intermediate between those of surface and interior are also present (Figure 18C). The interior of the colony has unbranched spindles. In the lobules the spindles are up to 2.5 mm long (Figure 16B), almost all having complex tubercles (Figure 16C). In the base of the colony they are up to 2 mm long (Figure 18A), many with complex tubercles (Figure 18B).

**Colour.** The alcohol-preserved specimen is brown.

#### Etymology.

Named after the late Prof. Lev Fishelson, Tel Aviv University, pioneering and outstanding marine biologist, who investigated Red Sea coral reefs.

#### Intraspecific variation.

The paratype ZMTAU Co 34138 (Figure 2E) has similar sclerites, colony shape and colour.

#### Remarks.

Preserved specimens have a brown colony colour. In the RMNH, only four specimens from Madagascar identified by Verseveldt as *Sinularia polydactyla* can be referred to this species. Live colonies are shown in Figure 44.

## Discussion

**Figure 19.**
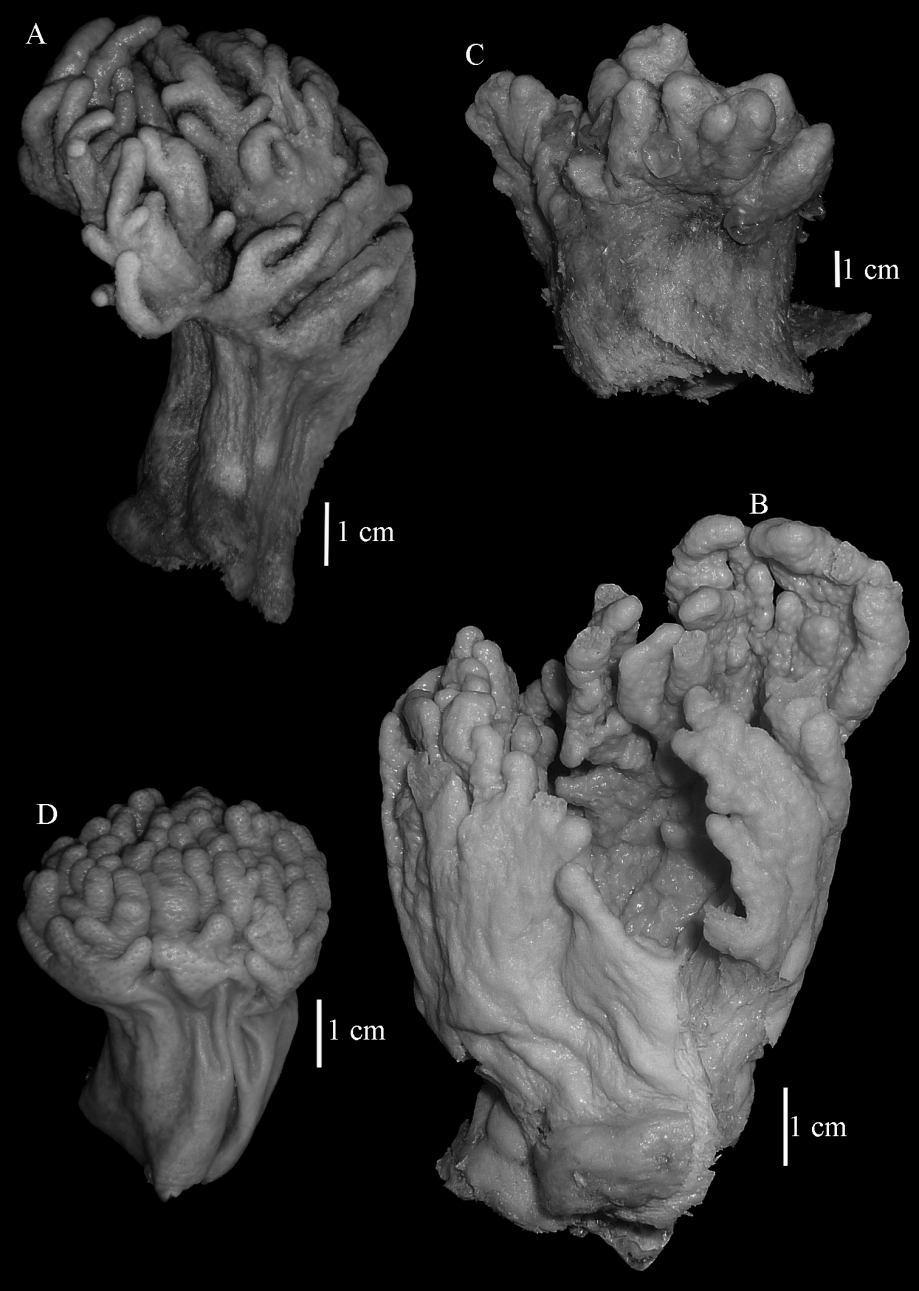
Colonies. **A** *Sinularia polydactyla*, RMNH 15950 **B** *Sinularia polydactyla*, ZMTAU Co 34181 **C** *Sinularia gibberosa*, ZMTAU 33611 **D** *Sinularia compressa*, RMNH 38420.

**Figure 20.**
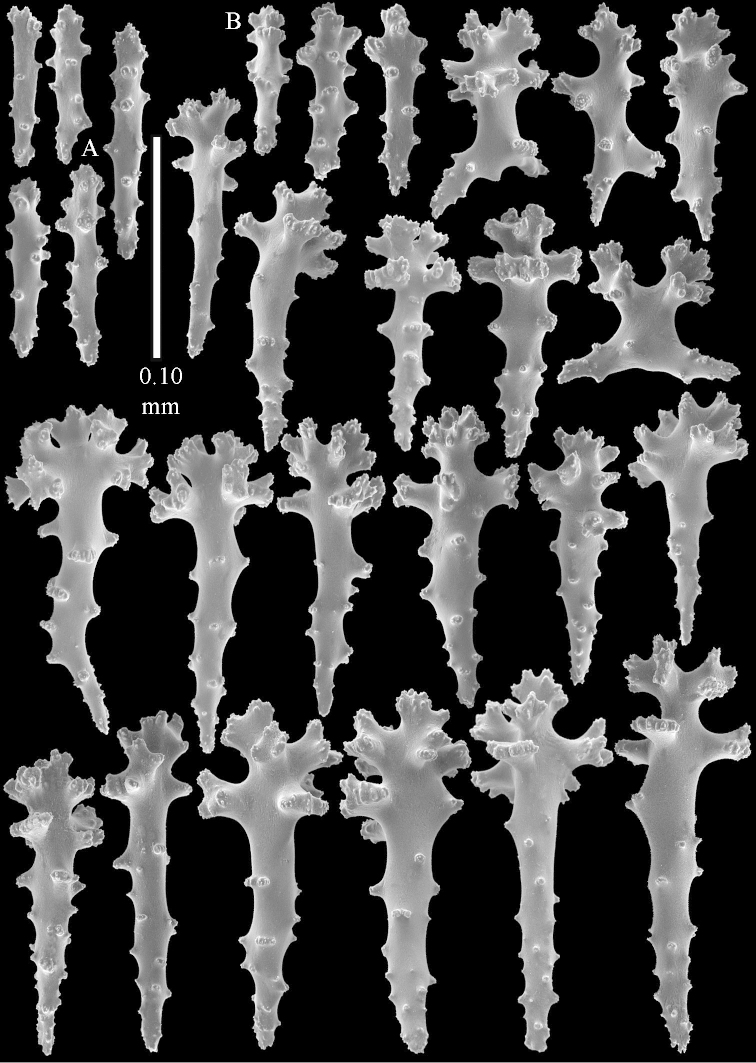
*Sinularia polydactyla* (Ehrenberg, 1834), RMNH 15950. **A** point clubs **B** clubs of surface layer top of colony.

**Figure 21.**
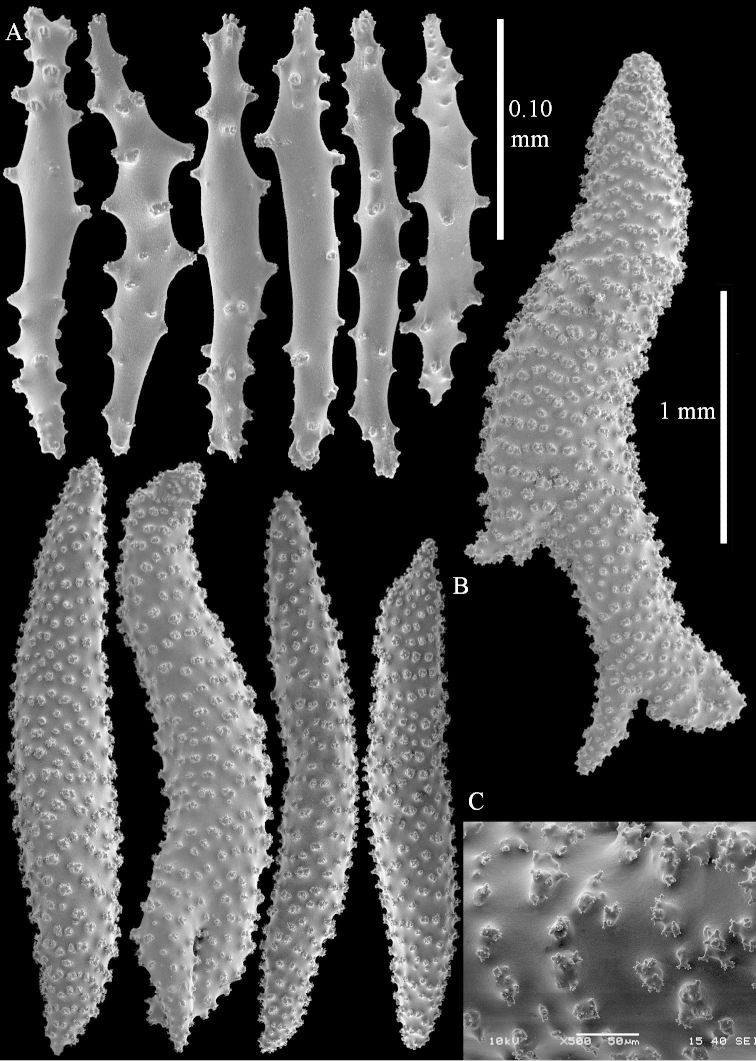
*Sinularia polydactyla* (Ehrenberg, 1834), RMNH 15950. **A** spindles of surface layer of top of colony **B** spindles of interior **C** tuberculation of a spindle.

**Figure 22.**
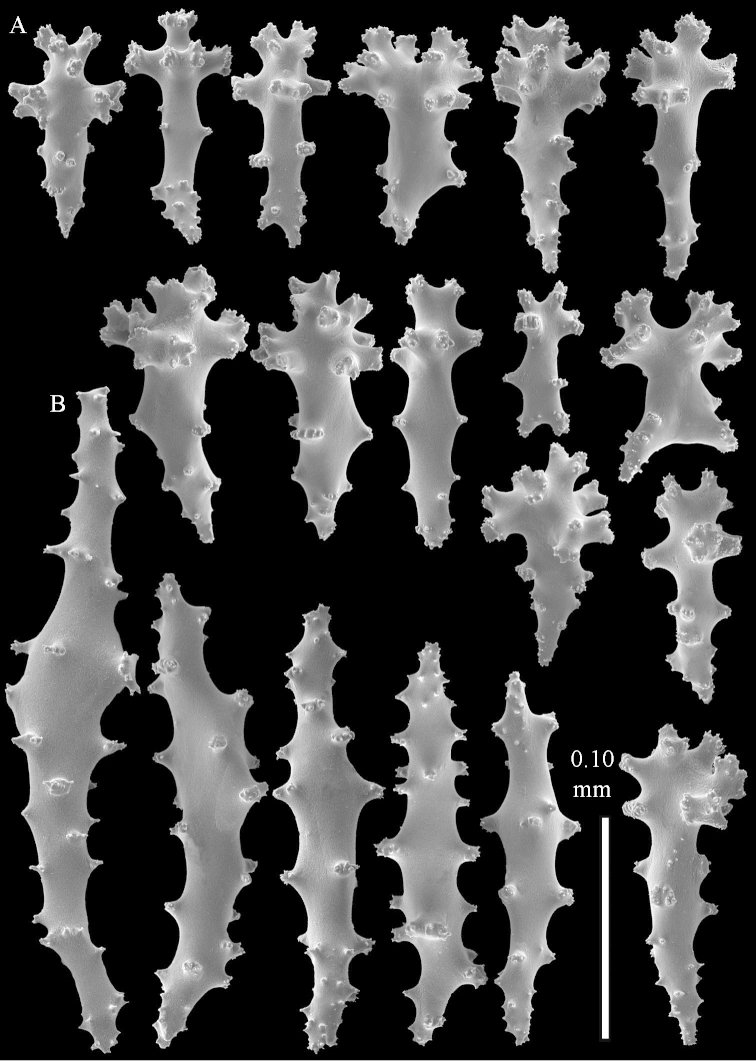
*Sinularia polydactyla* (Ehrenberg, 1834), RMNH 15950. **A** clubs of surface layer base of colony **B** spindles.

Material used by Verseveldt (1980: 108, fig. 57) to describe what he considered to represent *Sinularia polydactyla*, RMNH Coel. 15950 from Laing Island, Papua New Guinea was re-examined. Six specimens are present in the jar, but only one has clear signs of tissue sampling (Figure 19A) and therefore it must be the specimen studied by Verseveldt. The four microscope slides were claimed by Verseveldt to lack any polyp sclerites, but in the present study they proved to be clearly present (Figure 20A). These sclerites can be confused with the smallest clubs of the surface layer of the lobes (Figure 20B), but dissection of a single polyp of RMNH Coel. 15950 demonstrated that they are indeed derived from the polyps. Presence of polyp sclerites assigns the specimen to Clade 4B; in contrast, Ehrenberg’s lectotype (ZMB 299) lacks polyp sclerites, a character that assigns it to Clade 4D. This discrepancy suggests that Verseveldt’s identification of RMNH Coel. 15950 as *Sinularia polydactyla* was a mistake. Within Clade 4B the species that most closely resembles RMNH Coel. 15950 is *Sinularia sobolifera* Verseveldt & Tursch, 1979. Like Verseveldt’s *Sinularia polydactyla*, *Sinularia sobolifera* also was described from the Bismarck Sea, but from Mililat Bay. *Sinularia sobolifera* differs in having longer clubs, up to 0.27 mm long, with an almost smooth handle whereas the present material has clubs up to 0.18 mm long, with tuberculate handles. For completeness of the current study we depict the sclerites of the interior (Figure 21B–C) and base (Figure 22) of RMNH Coel. 15950.

**Figure 23.**
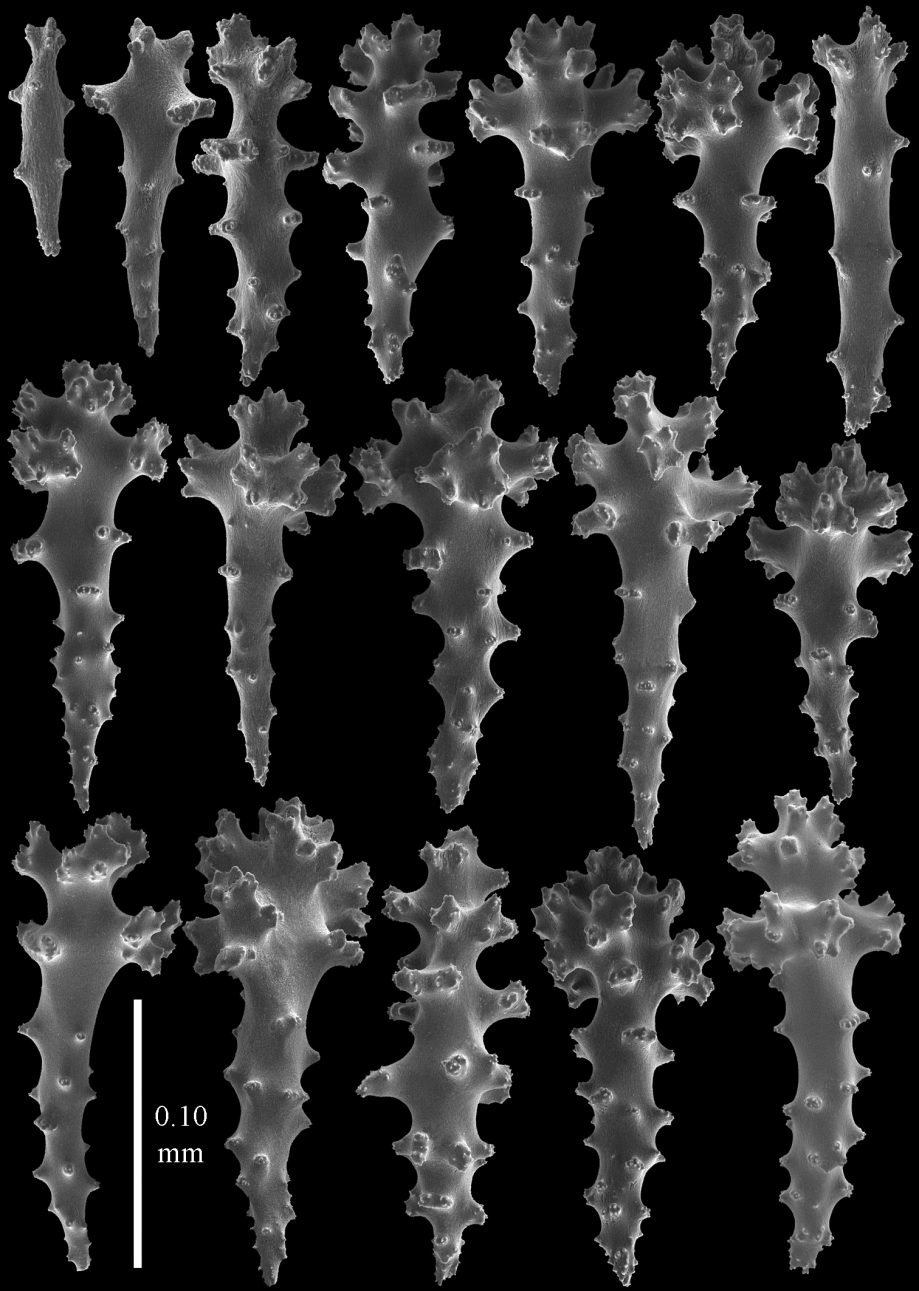
*Sinularia polydactyla* (Ehrenberg, 1834), ZMTAU Co 34181. Clubs of surface layer top of colony.

**Figure 24.**
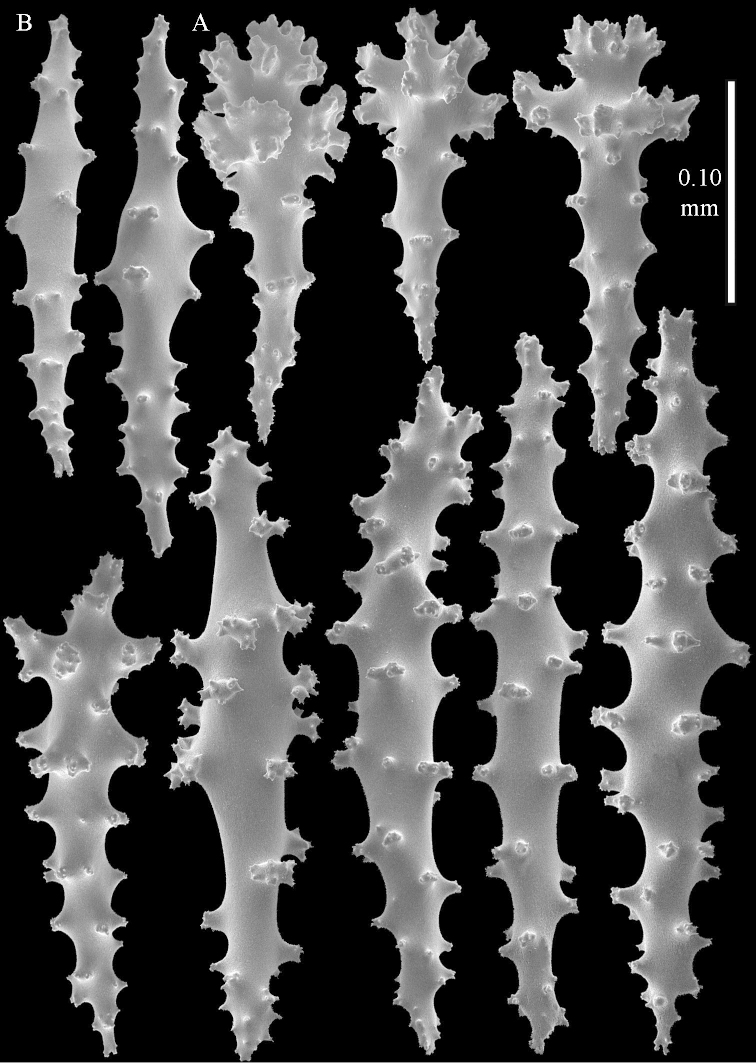
*Sinularia polydactyla* (Ehrenberg, 1834), ZMTAU Co 34181. **A** clubs of surface layer top of colony **B** spindles of surface layer top of colony.

**Figure 25.**
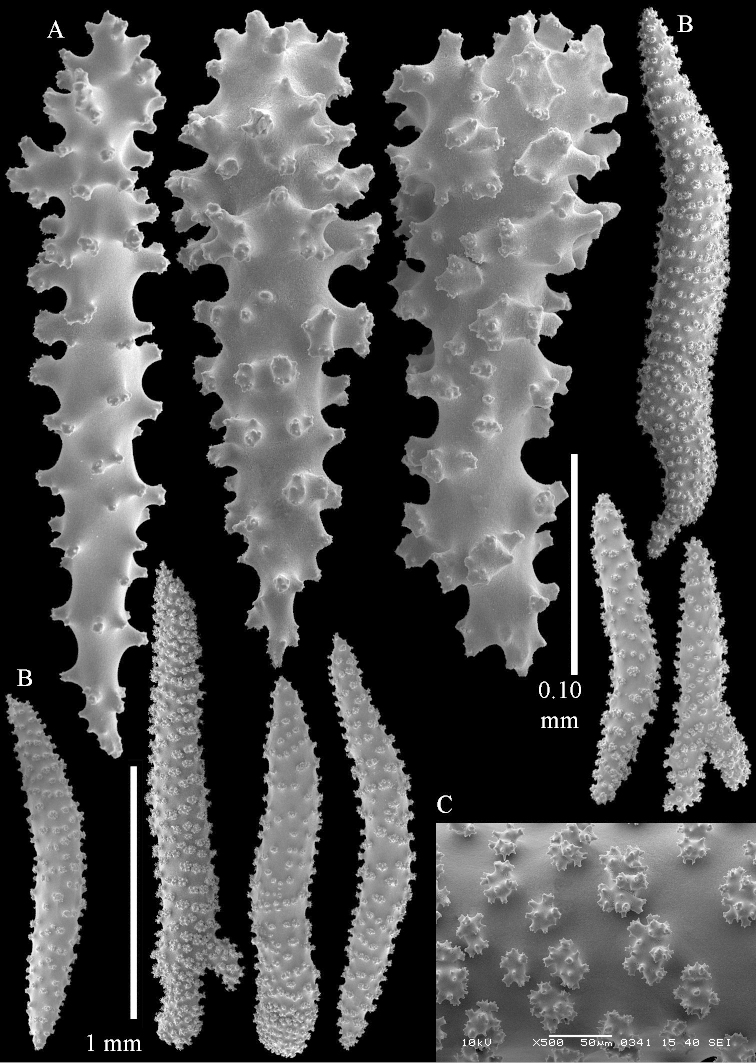
*Sinularia polydactyla* (Ehrenberg, 1834), ZMTAU Co 34181. **A** clubs of surface layer top of colony **B** spindles of interior of top of colony **C** tuberculation of a spindle.

**Figure 26.**
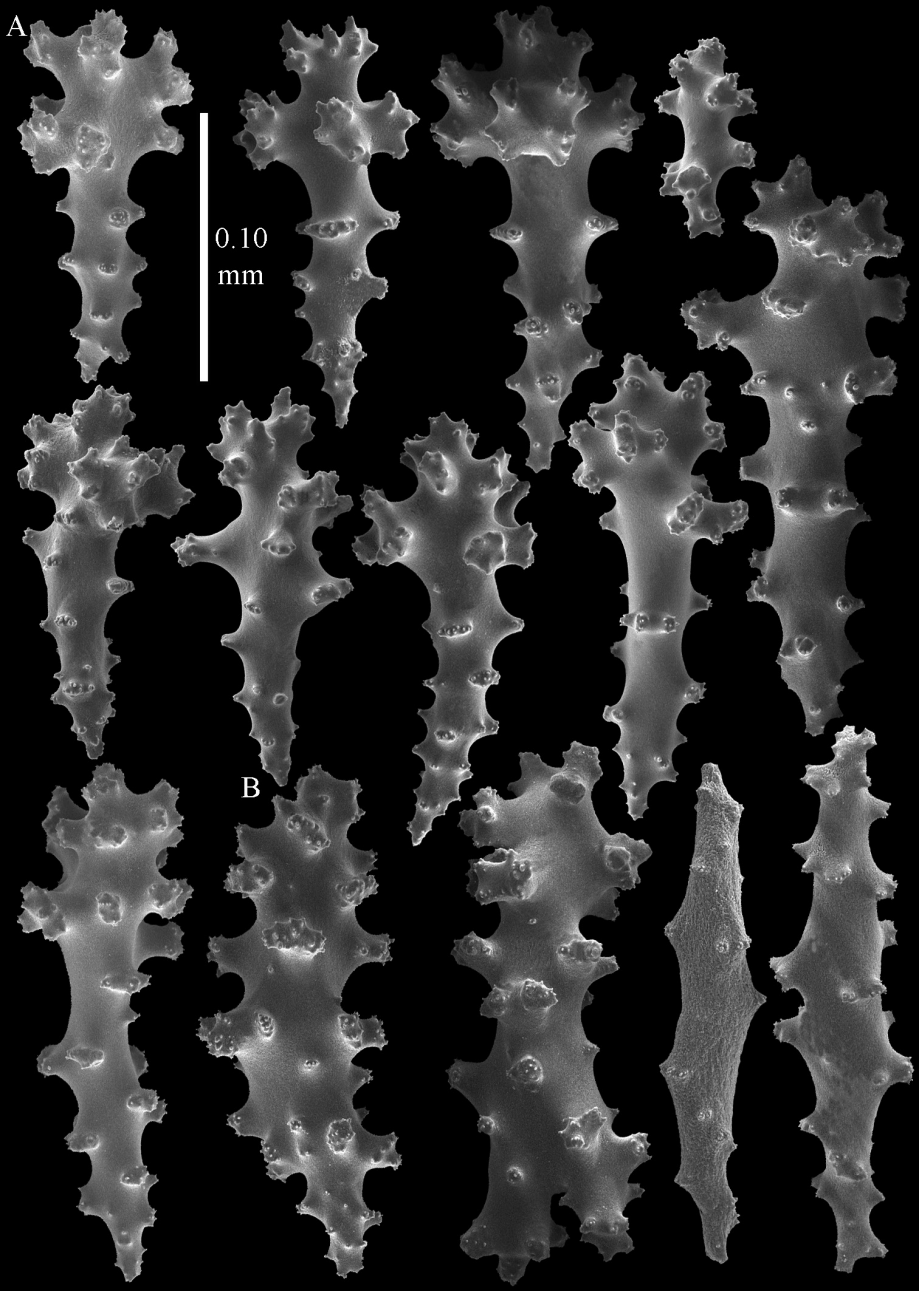
*Sinularia polydactyla* (Ehrenberg, 1834), ZMTAU Co 34181. **A** clubs of surface layer base of colony **B** spindles.

**Figure 27.**
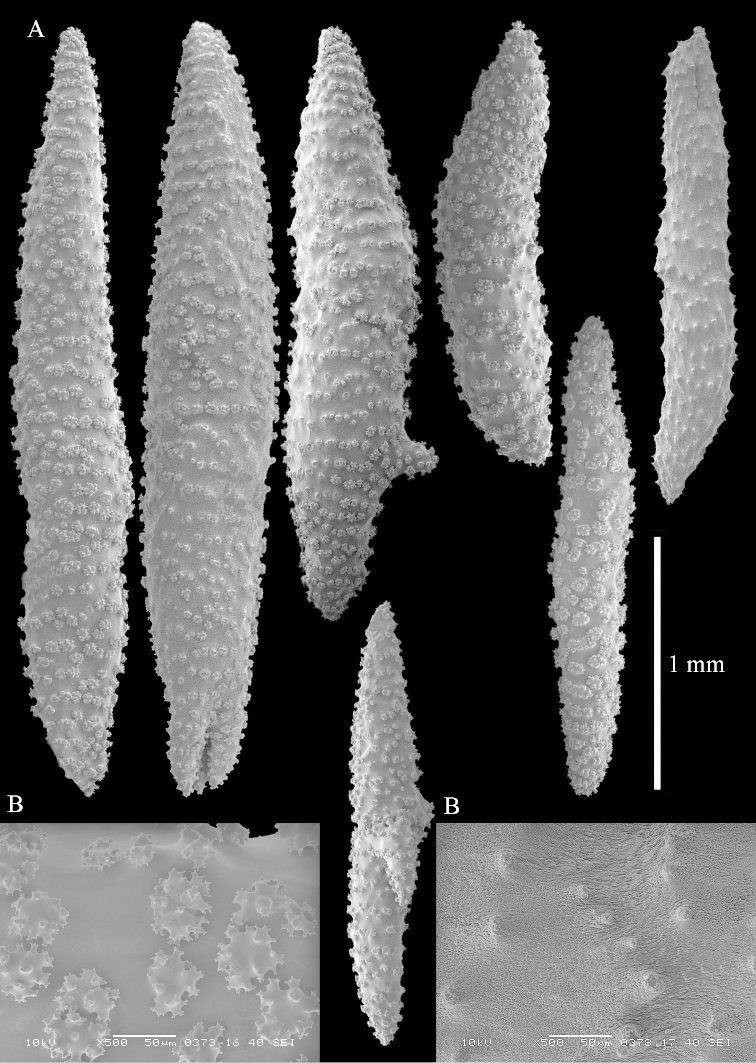
*Sinularia polydactyla* (Ehrenberg, 1834), ZMTAU Co 34181. **A** spindles of interior of base of colony **B** tuberculation of two of the spindles.

Both molecular and morphological evidence suggest that other specimens identified in the recent literature as *Sinularia polydactyla* or *Sinularia compressa* belong to neither of the two species described here, but instead represent either misidentifications or as yet undescribed species. ZMTAU Co 34181 (Israel, Gulf of Aqaba, Eilat, south Oil Jetty, 29°31.05'N, 34°55.86'E, 1.5 m, coll. Y. Benayahu, 25 July 2007), previously identified as *Sinularia polydactyla*, has a colony shape that differs from all other specimens examined, as it is not stalked but cup-shaped (Figure 19B). However, its sclerites do not differ much in shape from those of *Sinularia polydactyla* (Figures 23–27). This specimen is unique genetically, however, and its *mtMutS* sequence is unlike that of *Sinularia polydactyla* or any of the other reference species included in our analyses (Figure 1).

**Figure 28.**
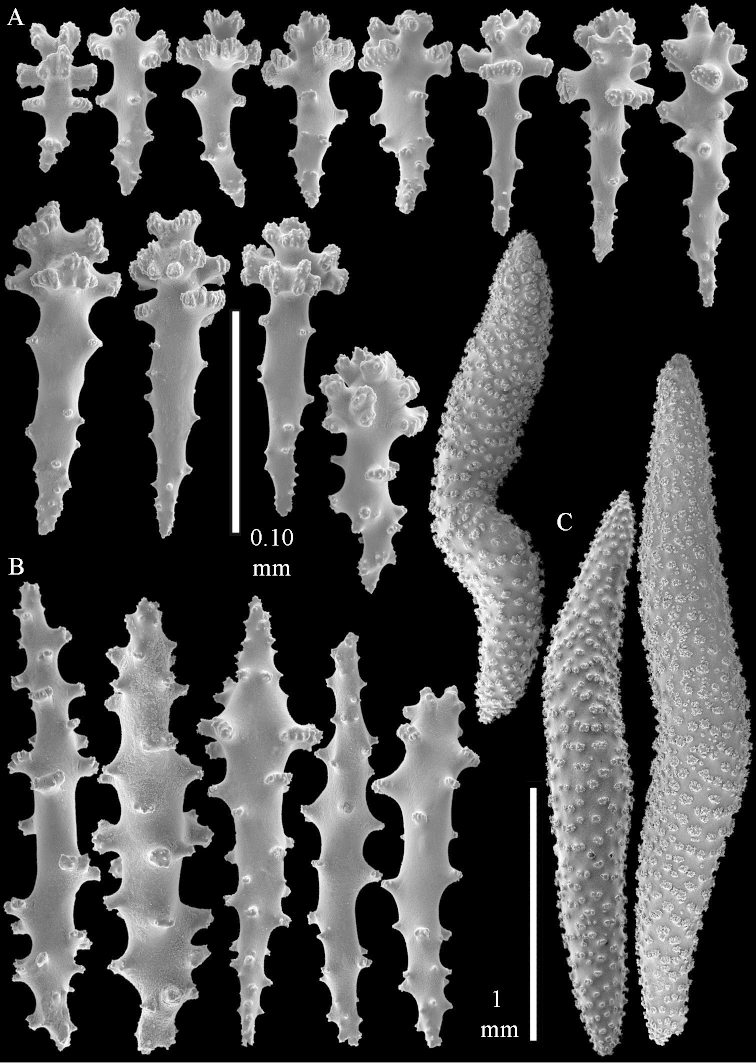
*Sinularia gibberosa* Tixier-Durivault, 1970, ZMTAU 33611. **A** clubs of surface layer base of colony **B** spindles **C** interior spindles.

**Figure 29.**
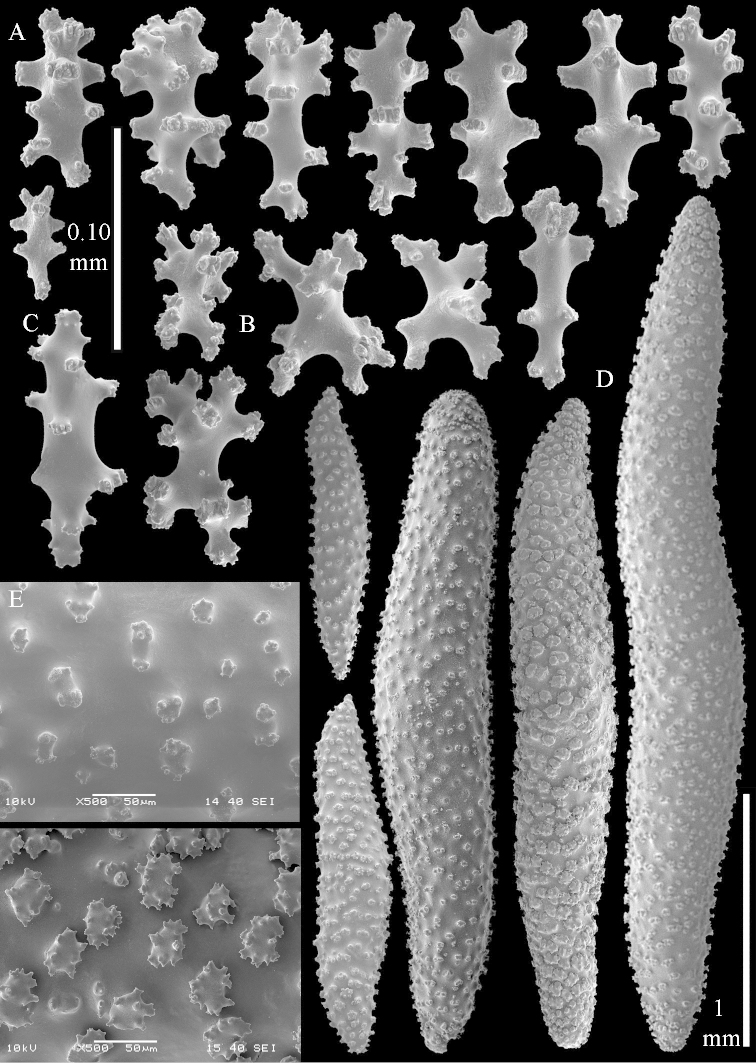
*Sinularia gibberosa* Tixier-Durivault, 1970, ZMTAU 33611. **A** clubs of surface layer base of colony **B** crosses **C** spindle **D** interior spindles **E** tuberculation of spindles.

**Figure 30.**
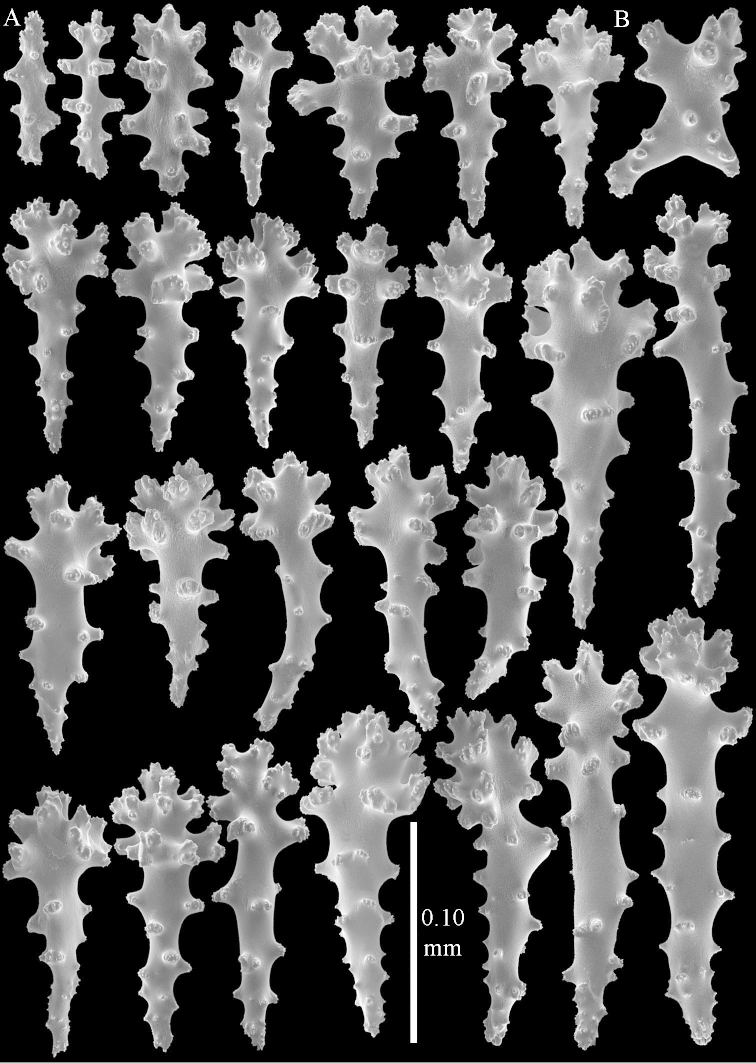
*Sinularia compressa* Tixier-Durivault, 1945, RMNH Coel. 38420. **A** clubs of surface layer top of colony **B** cross.

**Figure 31.**
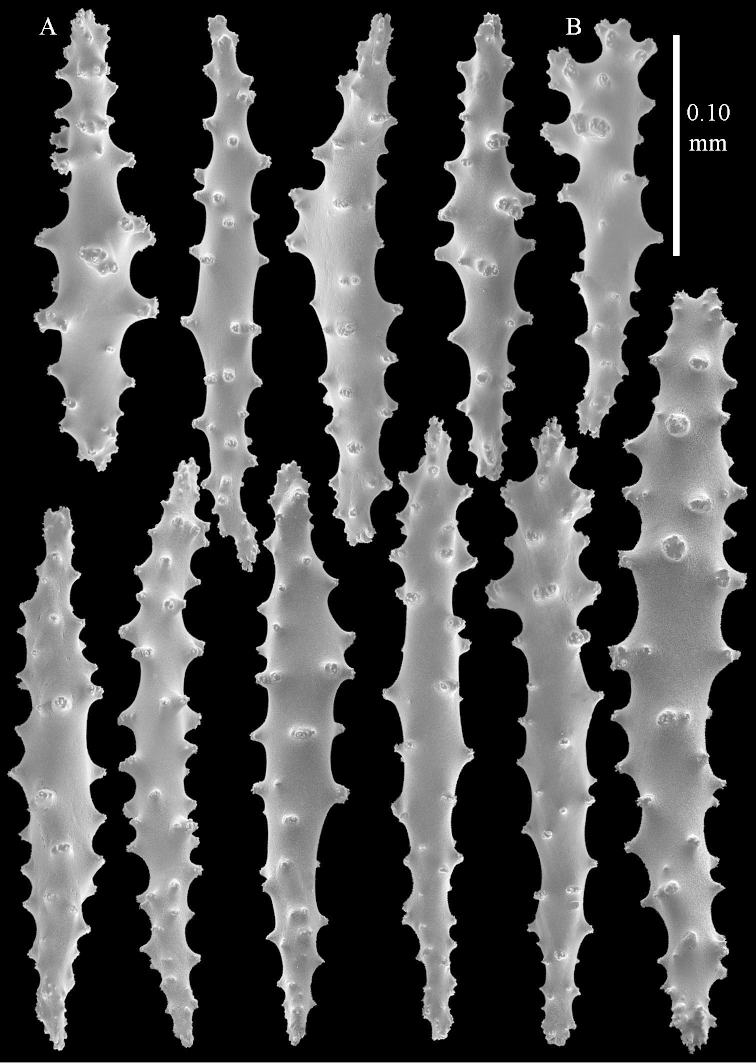
*Sinularia compressa* Tixier-Durivault, 1945, RMNH Coel. 38420. **A** spindles of surface layer of top of colony **B** club.

**Figure 32.**
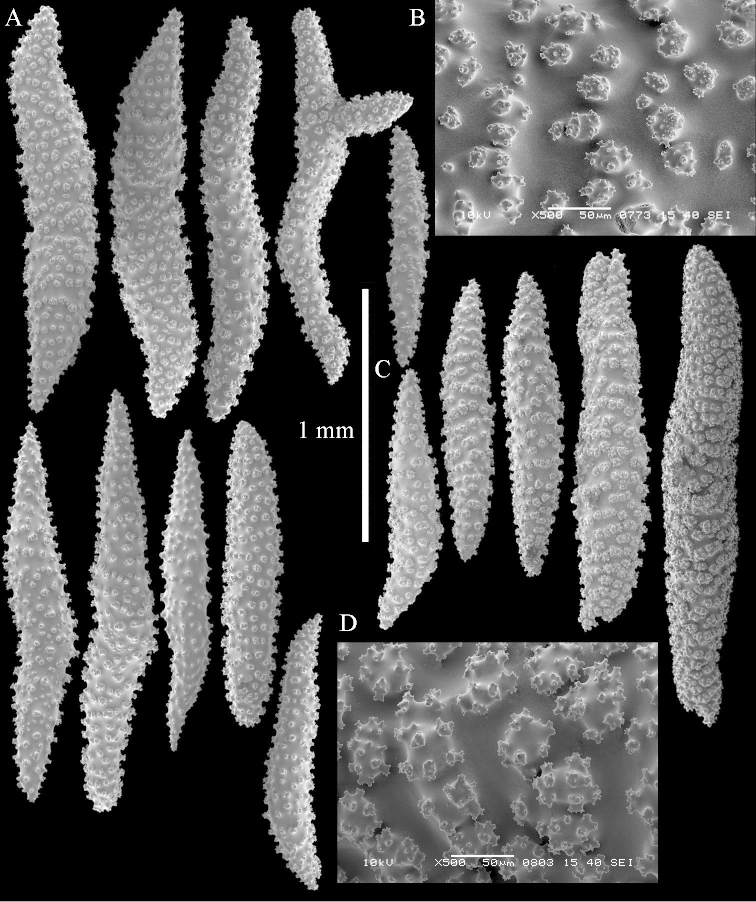
*Sinularia compressa* Tixier-Durivault, 1945, RMNH Coel. 38420. **A** spindles of interior of top of colony **B** tuberculation of one of the spindles **C** spindles of the interior of the base of the colony **D** tuberculation of one of the spindles.

**Figure 33.**
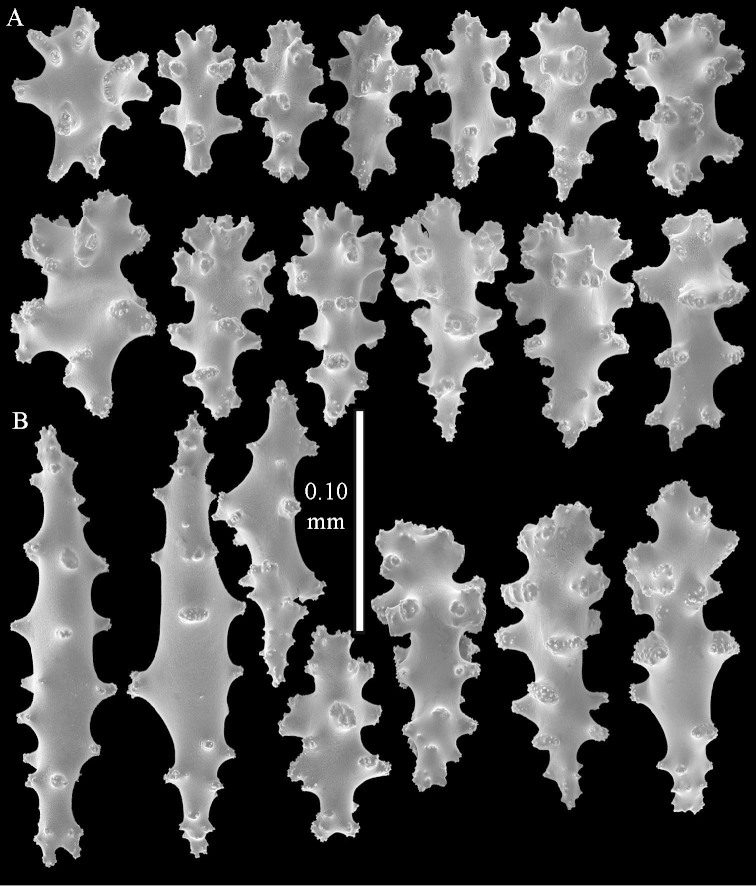
*Sinularia compressa* Tixier-Durivault, 1945, RMNH Coel. 38420. **A** clubs of surface layer base of colony **B** four spindles.

**Figure 34.**
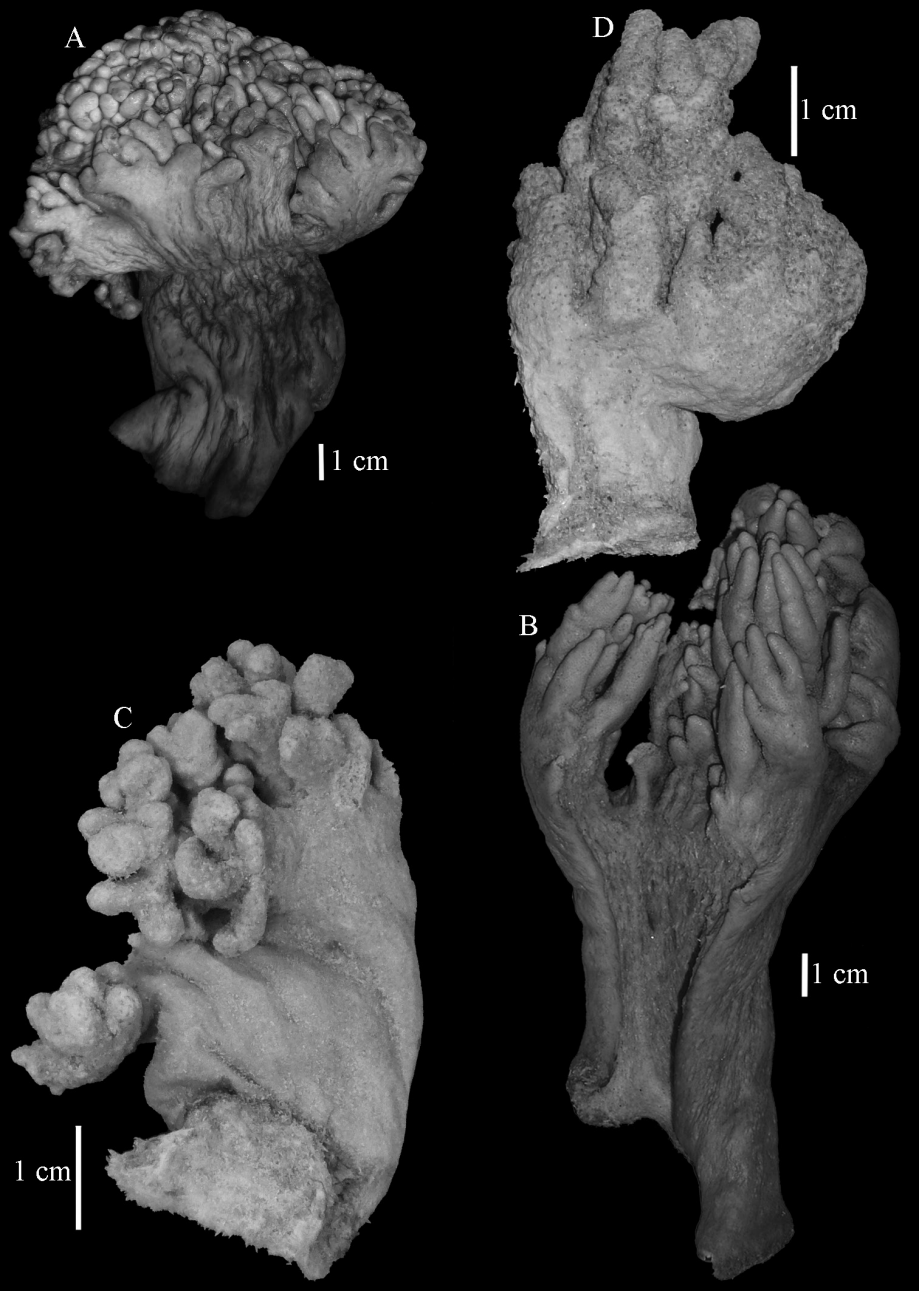
Colonies. A *Sinularia polydactyla*, RMNH 19566 **B** *Sinularia ceramensis*, RMNH 38442 **C** *Sinularia polydactyla*, “PBH-Tr3” **D** *Sinularia polydactyla*, “PBH-C10”.

**Figure 35.**
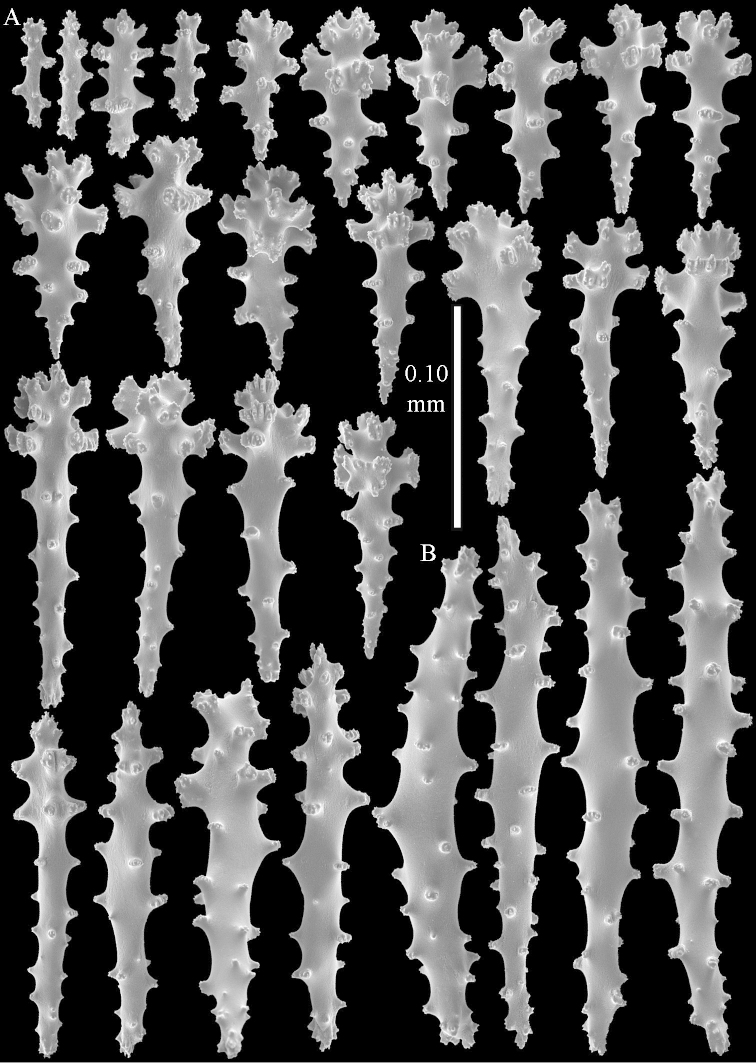
*Sinularia polydactyla* Tixier-Durivault, 1945, RMNH Coel. 19566. **A** clubs of surface layer top of colony **B** spindles.

Three of the nine specimens from the western Pacific that belonged to a well-supported clade within Clade 4D were not re-examined in the current study: NTM C14142 (*Sinularia polydactyla*, American Samoa), NTM C14173 (*Sinularia polydactyla*, Papua New Guinea), and RMNH Coel. 41339 (*Sinularia polydactyla*, Palau); the first two were not available and the third was a very small fragment. The other specimens belonging to this western Pacific sub-clade have been re-examined. ZMTAU Co 33611 (*Sinularia gibberosa* Tixier-Durivault, 1970) was also re-examined as its *mtMutS* (*msh1*) sequence placed it among specimens of *Sinularia polydactyla* in Benayahu et al. (2013: 1544). The colony of ZMTAU Co 33611, shown in Figure 19C, is somewhat different from the normal colony shape of *Sinularia gibberosa* (Verseveldt, 1980: pls. 17-18). However, its sclerites (Figures 28–29) very much resemble those of *Sinularia gibberosa* and therefore the original identification is maintained in the present study despite the fact that more recently obtained sequences from specimens identified as *Sinularia gibberosa* do not match this one (unpublished data). RMNH Coel. 38420 (*Sinularia compressa*, Ambon) has a much longer stalk (Figure 19D) than commonly found in *Sinularia polydactyla* and *Sinularia compressa* although no distinct differences among the sclerites of these species were shown (Figures 30–33). It is genetically different from the Red Sea *Sinularia compressa* specimens under study, and therefore it is considered a misidentification and probably represents an as yet undescribed species. RMNH Coel. 19566 from Ambon (Figure 34A), identified by Ofwegen and Vennam (1994) as *Sinularia polydactyla*, was also re-examined (Figure 35) and found to be close to RMNH Coel. 38420.

**Figure 36.**
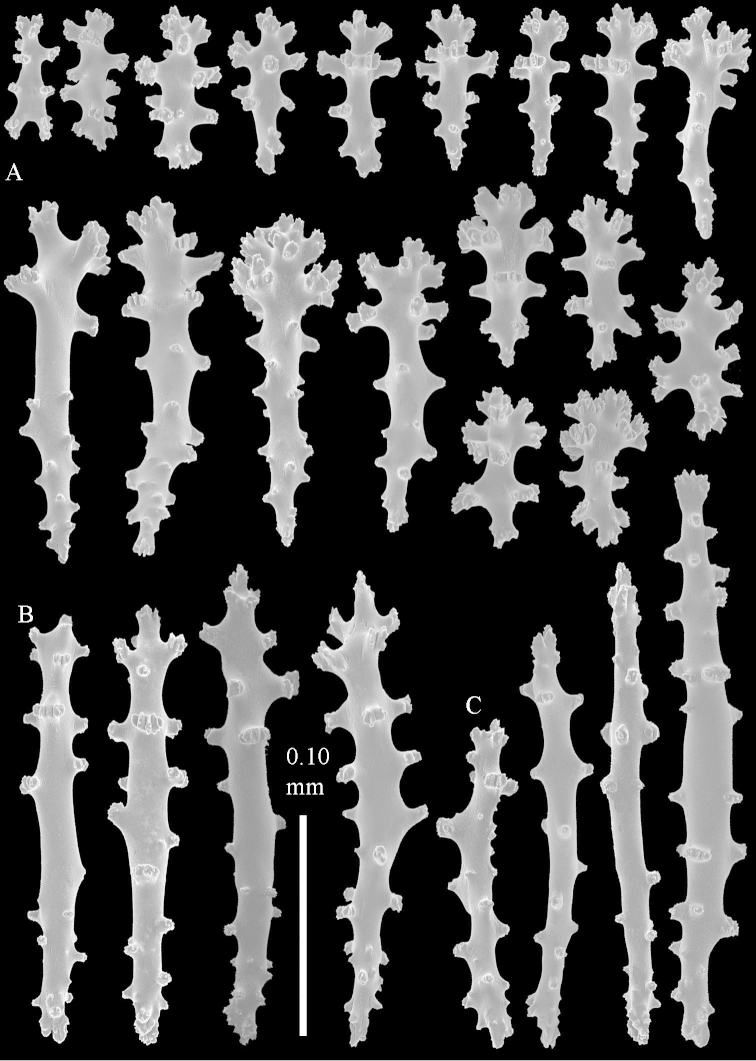
*Sinularia ceramensis* Verseveldt, 1977, RMNH 38442. **A** clubs of surface layer top of colony **B** intermediates between clubs and spindles **C** spindles.

**Figure 37.**
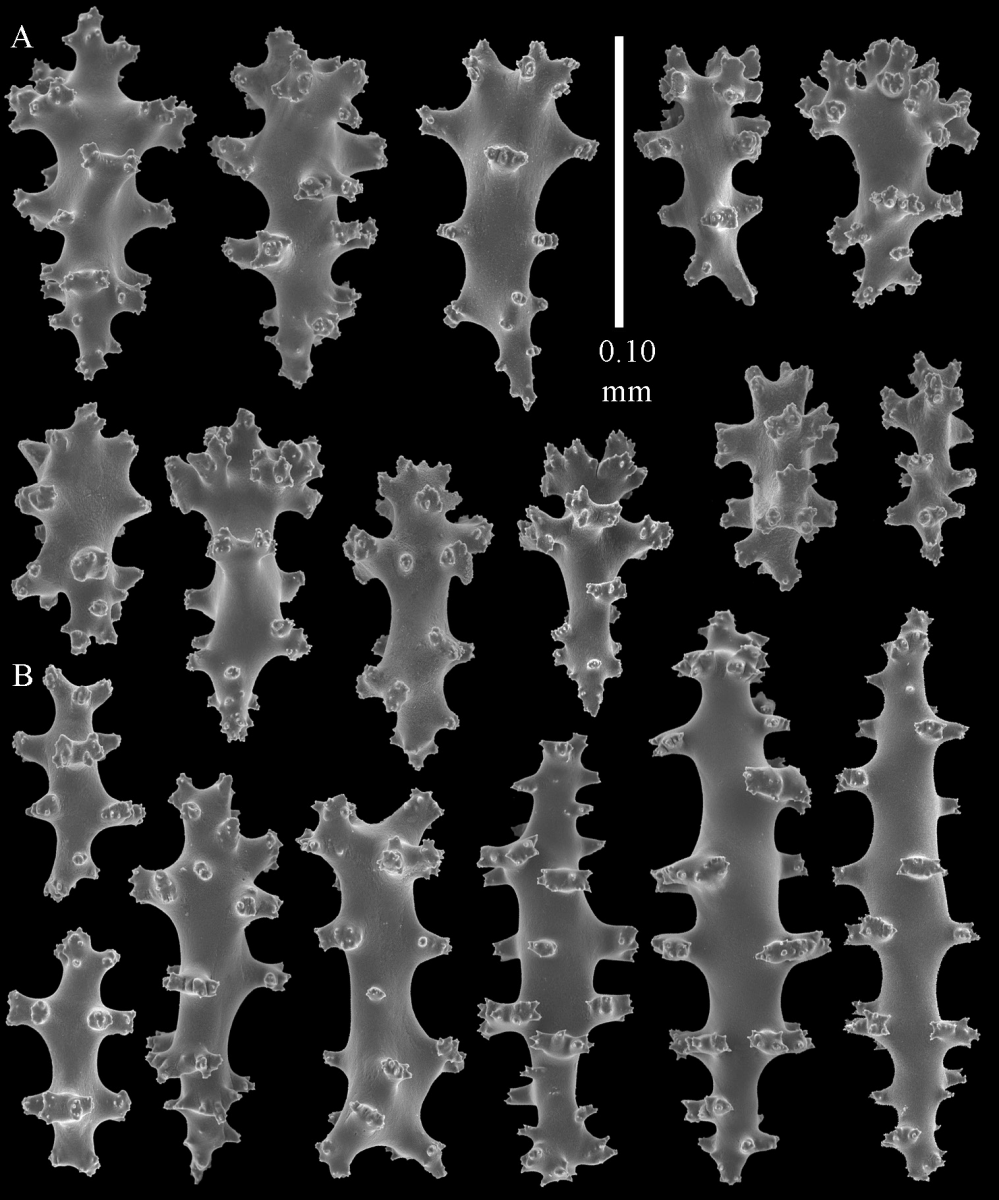
*Sinularia ceramensis* Verseveldt, 1977, RMNH 38442. **A** clubs of surface layer base of colony **B** spindles.

RMNH Coel. 38442 (*Sinularia polydactyla*, Ambon) is now considered and assigned, with some doubts, to *Sinularia ceramensis* Verseveldt, 1977. Verseveldt described that species with the following characters: lobes up to 4 cm high, flattened; surface layer of coenenchyme with clubs with a central wart, 0.06–0.09 mm long, some up to 0.14 mm; small spindles, 0.15–0.25 mm long, with simple tubercles. Stalk surface with wider clubs. Interior with pointed and blunt-ended spindles up to 3 mm long, with a median constriction and covered with medium sized warts. RMNH Coel. 38442 differs from this in having long tapering lobes (Figure 34B), and many of the clubs with a central wart are up to 0.15 mm long (Figure 36A). In addition, some intermediates (Fig. 36B) between small spindles (Figure 36C) and clubs, even up to 0.20 mm long are also found. The surface of the base shows shorter and wider sclerites, many with tubercles with acute ends (Figure 37), which were not reported for *Sinularia ceramensis*. Because of these differences and the previously unknown colony shape of *Sinularia polydactyla*, this specimen was originally identified as that species.

**Figure 38.**
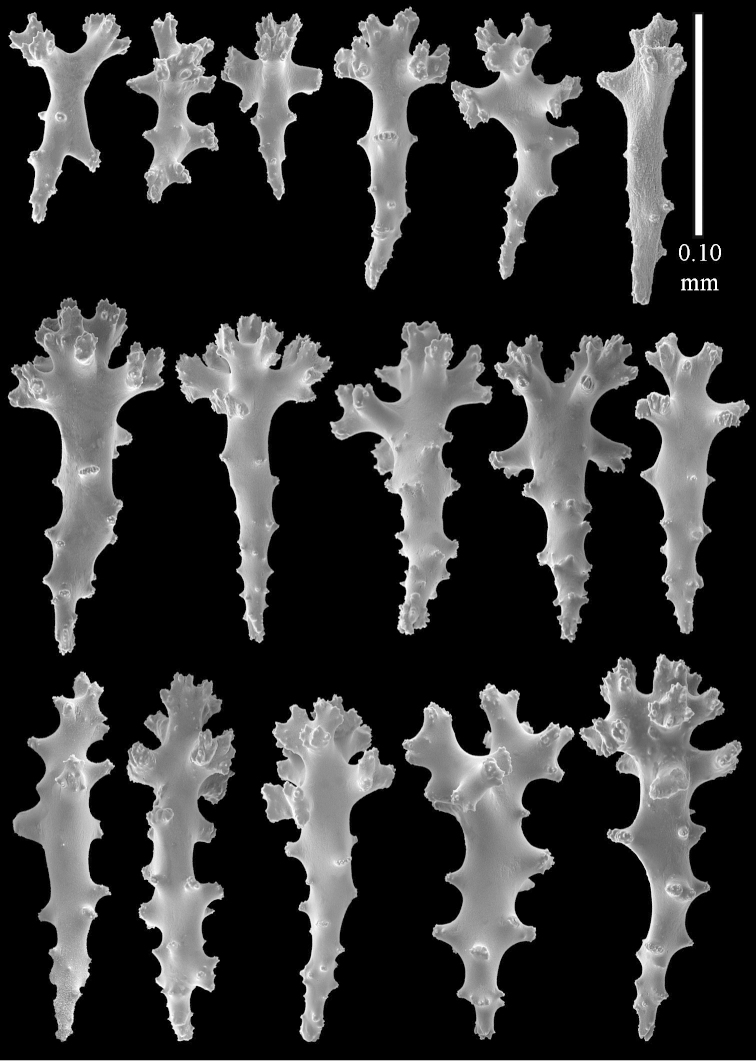
*Sinularia polydactyla*, “PBH-Tr3”. Clubs of surface layer top of colony.

**Figure 39.**
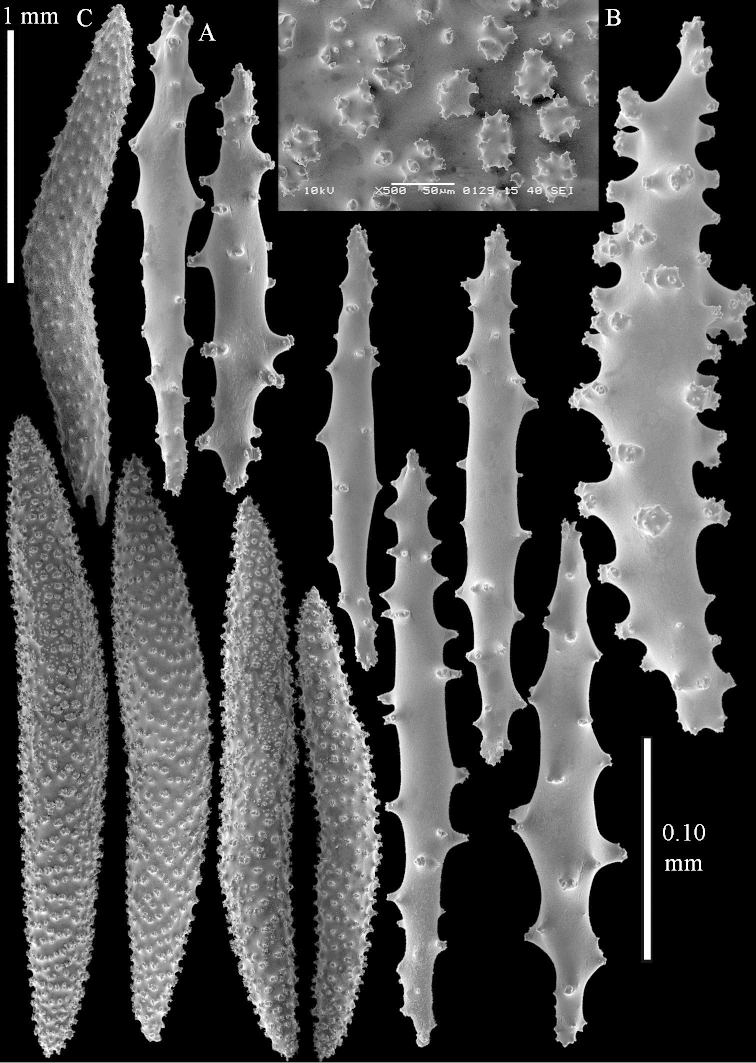
*Sinularia polydactyla*, “PBH-Tr3”. **A** spindles of surface layer top of colony **B** tuberculation of one of the spindles **C** spindles of the interior of the top of the colony.

**Figure 40.**
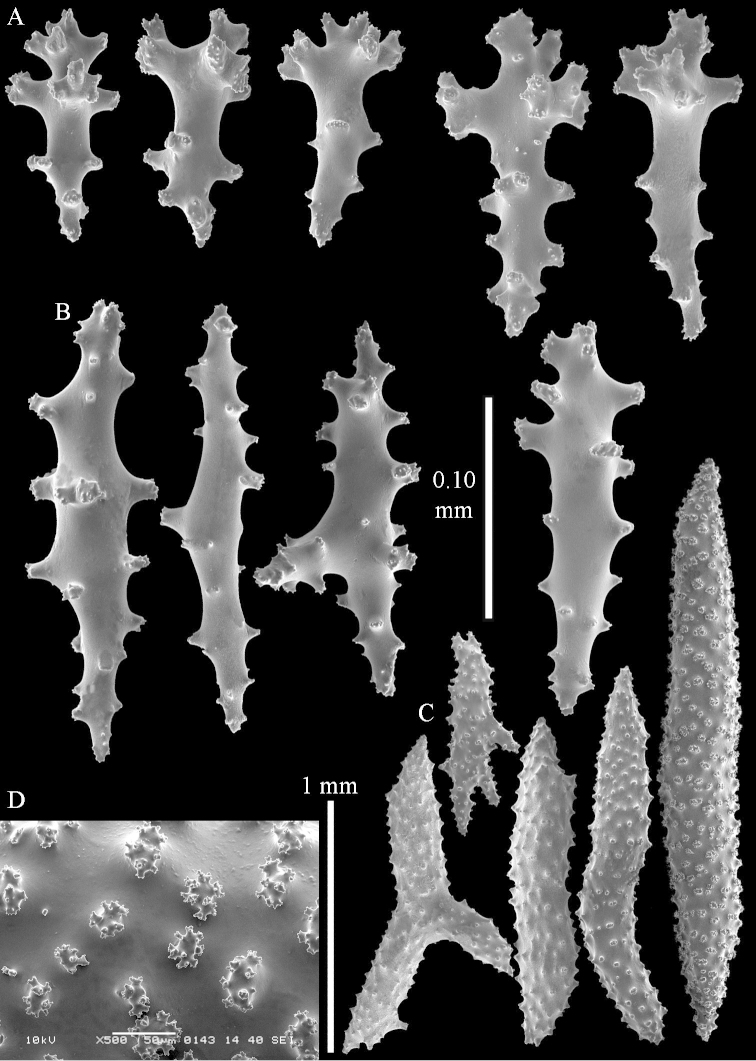
*Sinularia polydactyla*, “PBH-Tr3”. **A** clubs of surface layer base of colony **B** spindles **C** spindles of the interior of the base of the colony **D** tuberculation of one of the spindles.

**Figure 41.**
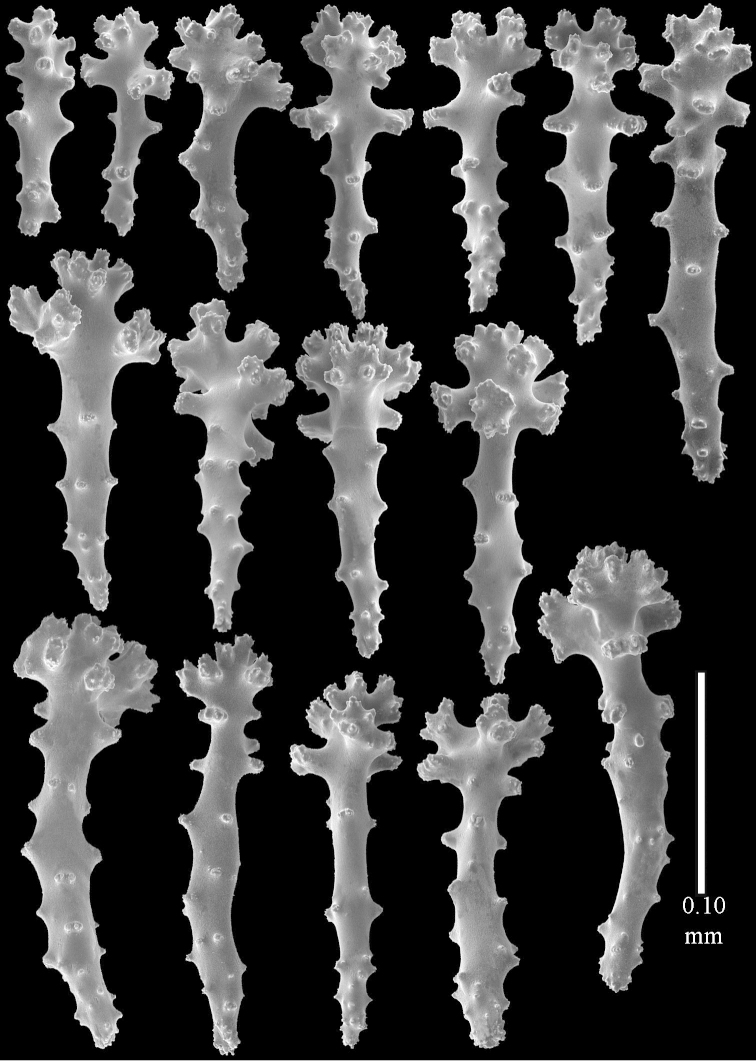
*Sinularia polydactyla*, “PBH-C10”. Clubs of surface layer top of colony.

**Figure 42.**
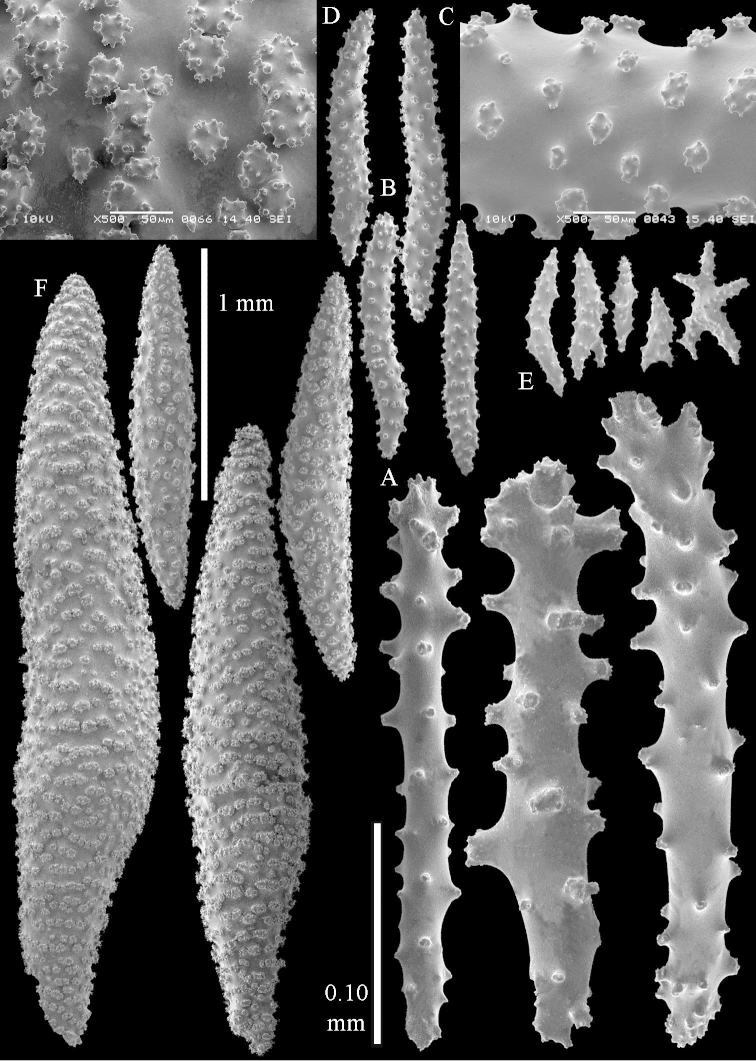
*Sinularia polydactyla*, “PBH-C10”. **A** clubs of surface layer top of colony **B** spindles of the interior of the top of the colony **C** tuberculation of one of the spindles of the interior of the top of the colony **D** tuberculation of one of the spindles of the interior of the base of the colony **E-F** spindles of the interior of the base of the colony.

**Figure 43.**
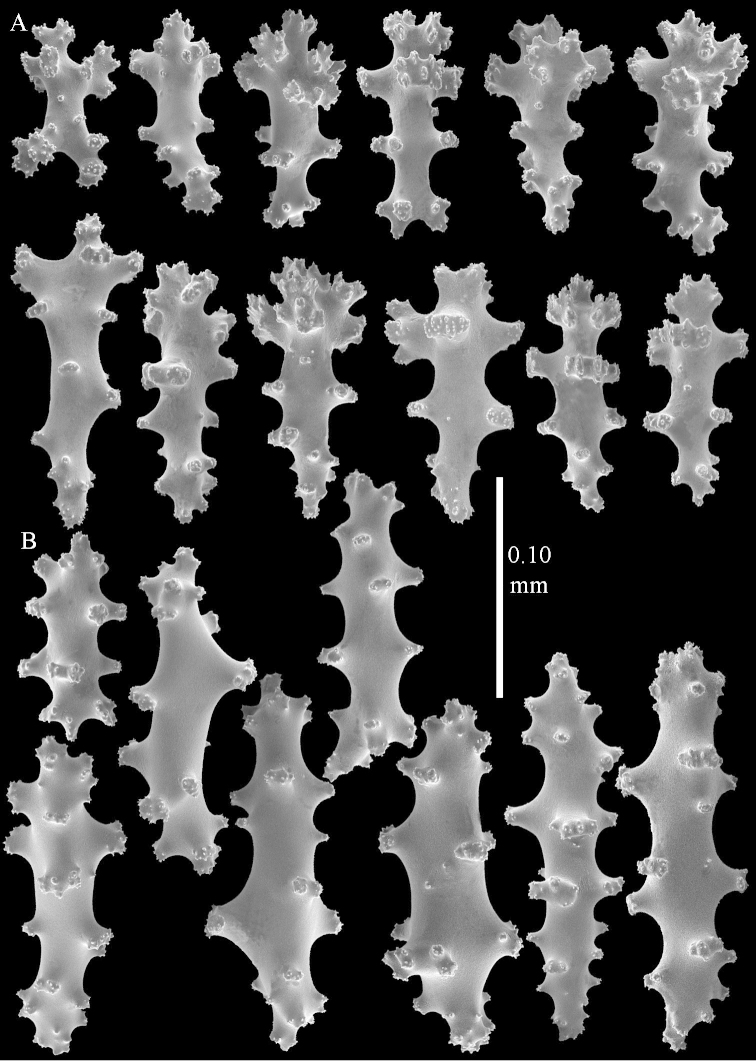
*Sinularia polydactyla*, “PBH-C10”. **A** clubs of surface layer base of colony **B** spindles.

Material used by Hoover et al. (2008) (*Sinularia polydactyla*, Guam) was also re-examined as their specimens identified as *Sinularia polydactyla* appeared to belong to three different species in the phylogenetic tree (Figure 1). Their three “PBH-To” fragments did not contain any sclerites, probably because the material was preserved using a chemical (RNAlater, Ambion Inc.) that dissolves sclerites. Their *Sinularia polydactyla* specimens “PBH-Tr3”, and “PBH-C6” and “PBH-C10” formed a sub-clade with *Sinularia nanolobata* Verseveldt, 1977 and *Sinularia scabra* Tixier-Durivault, 1970 (Fig. 1). The “PBH-Tr3” specimen (Figure 34C) featured sclerites (Figures 38–40) that are quite different from *Sinularia polydactyla* but did not match any other *Sinularia* species known at present. The club-shaped sclerites of “PBH-C10” (Figures 41, 42A, 43A) resemble those of *Sinularia scabra*, the sister taxon in the phylogenetic tree, as does its colony shape (Figure 34D). The internal spindles (Figure 42B-F) are however quite different from those described by Verseveldt (1980). It showed much smaller spindles in the lobes (Figure 42B) and many small branched spindles in the colony base (Figure 42E) that were not reported by Verseveldt (1980). Despite these differences “PBH-C10” is presently considered to belong to *Sinularia scabra*. Notably, “PBH-C6” did not differ much from “PBH-C10”. The three dry specimens discussed above are in poor condition and not suitable for a formal taxonomic description.

**Figure 44.**
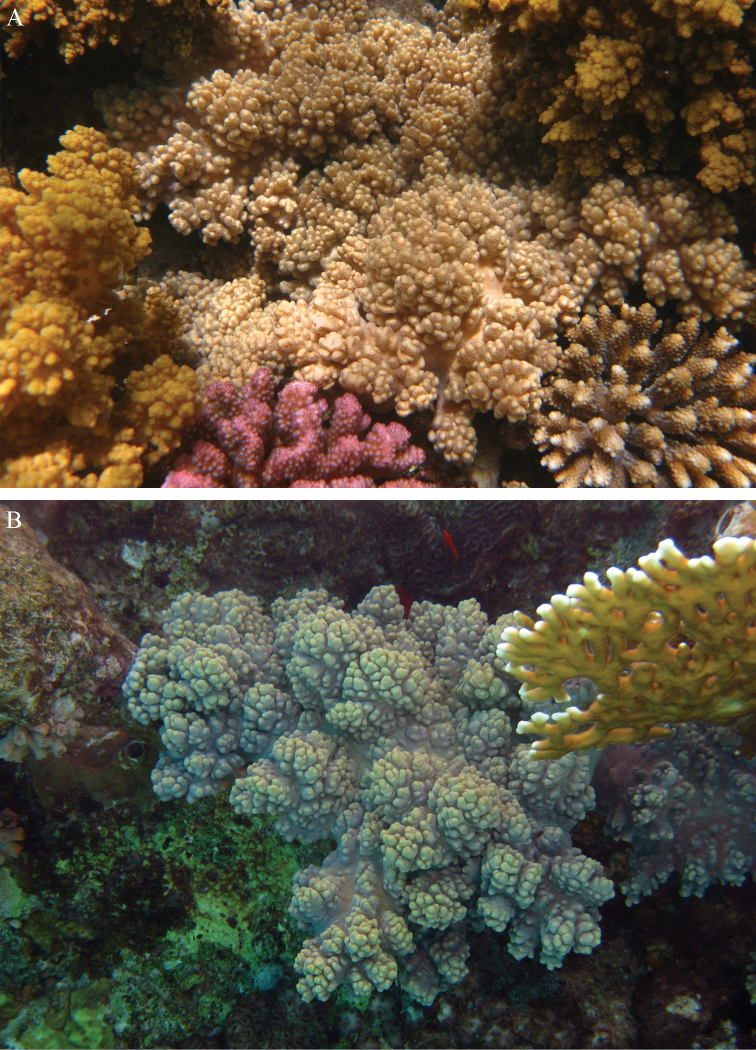
Live colonies *Sinularia levi* sp. n. **A** ZMTAU 36585 **B** ZMTAU 36607. Photographs by Erez Shoham.

## Supplementary Material

XML Treatment for Sinularia polydactyla

XML Treatment for Sinularia levi

## Supplementary materials

Supplementary material 1 Maximum likelihood phylogeny Data type: Figure Explanation note: Maximum likelihood phylogeny of *Sinularia* clade 4 based on a combined, partitioned analysis of mtMutS (735 bp) and COI + igr1 (815 bp). Numbers above branches are ML bootstrap percentages; numbers below branches are posterior probabilities from Bayesian Inference. Red: specimens identified in previous work as *Sinularia polydactyla*; blue: specimens identified in previous work as *Sinularia compressa*. This dataset is made available under the Open Database License (http://opendatacommons.org/licenses/odbl/1.0/). The Open Database License (ODbL) is a license agreement intended to allow users to freely share, modify, and use this Dataset while maintaining this same freedom for others, provided that the original source and author(s) are credited.Leen P. van Ofwegen, Catherine S. McFadden, Yehuda Benayahu

Supplementary material 2 GenBank accession numbers Data type: occurence Explanation note: Specimens of *Sinularia* included in molecular phylogenetic analysis. NTM = Museum and Art Gallery of the Northern Territory ; RMNH = Naturalis Biodiversity Center (Rijksmuseum, Leiden) ; ZMTAU = Zoological Museum, Tel Aviv University ; UF = Florida Natural History Museum ; USNM = Smithsonian Institution . NA = no sequence available . This dataset is made available under the Open Database License (http://opendatacommons.org/licenses/odbl/1.0/). The Open Database License (ODbL) is a license agreement intended to allow users to freely share, modify, and use this Dataset while maintaining this same freedom for others, provided that the original source and author(s) are credited.Leen P. van Ofwegen, Catherine S. McFadden, Yehuda Benayahu
