# Supplementary material for: Sinularia polydactyla (Ehrenberg, 1834) (Cnidaria, Octocorallia) re-examined, with the description of a new species
Source: Zookeys. 2016 Apr 14;(581):71–126. doi: 10.3897/zookeys.581.7455 (PMC4857042; doi:10.3897/zookeys.581.7455)
Supplement: Supplementary material 1 — Maximum likelihood phylogeny [file zookeys-581-071-s001.pdf]

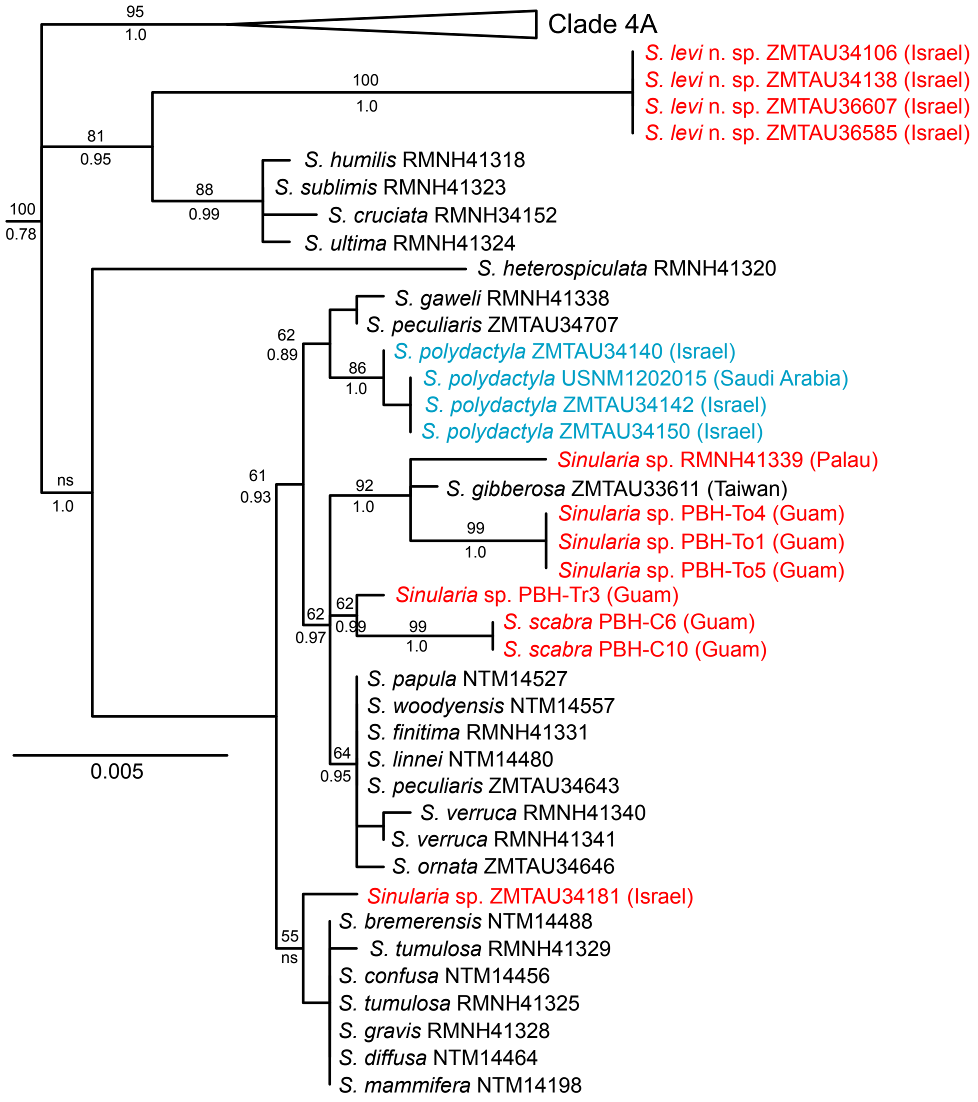

Supplemental Figure 1: Maximum likelihood phylogeny of *Sinularia* clade 4 based on a combined, partitioned analysis of mtMutS (735 bp) and COI + igr1 (815 bp). Numbers above branches are ML bootstrap percentages; numbers below branches are posterior probabilities from Bayesian Inference. Red: specimens identified in previous work as *S. polydactyla*; blue: specimens identified in previous work as *S. compressa*.
