# Supplementary material for: Sinularia polydactyla (Ehrenberg, 1834) (Cnidaria, Octocorallia) re-examined, with the description of a new species
Source: Zookeys. 2016 Apr 14;(581):71–126. doi: 10.3897/zookeys.581.7455 (PMC4857042; doi:10.3897/zookeys.581.7455)
Supplement: Supplementary material 2 — GenBank accession numbers [file zookeys-581-071-s002.docx]

Supplementary Table S1: Specimens of *Sinularia* included in molecular phylogenetic analysis. NTM = Museum and Art Gallery of the Northern Territory; RMNH = Naturalis Biodiversity Center (was Rijksmuseum van Natuurlijke Historie, Leiden); ZMTAU = Zoological Museum, Tel Aviv University; UF = Florida Natural History Museum; USNM = National Museum of Natural History, Smithsonian Institution, Washington, D.C. (was United States National Museum). NA = no sequence available.

| **Species** | **Museum Cat. No.** | **Collection Location** | **GenBank Acc. #** | |
| --- | --- | --- | --- | --- |
|  |  |  | **mtMutS** | **COI** |
| *S. abhishiktae* | RMNH Coel. 38720 | Palau | FJ621373 | NA |
| *S. babeldaobensis* | RMNH Coel. 38723 | Palau | FJ621377 | NA |
| *S. bremerensis* | NTM C14488 | N. Australia | FJ621382 | KU230375 |
| *S. capitalis* | NTM C14530 | N. Australia | FJ621383 | NA |
| *S. ceramensis* | RMNH Coel. 38442 | Indonesia, Ambon | FJ621464 | NA |
| *S. ?ceramensis* | RMNH Coel. 38420 | Indonesia, Ambon | FJ621385 | NA |
| *S. confusa* | NTM C14456 | N. Australia | FJ621390 | KU230376 |
| *S. crassa* | RMNH Coel. 38430 | Indonesia, Ambon | FJ621391 | NA |
| *S. crebra* | RMNH Coel. 38726 | Palau | FJ621392 | NA |
| *S. cruciata* | ZMTAU Co 34152 | Red Sea, Israel | FJ621395 | GU355988 |
| *S. curvata* | RMNH Coel. 38436 | Indonesia, Ambon | FJ621396 | NA |
| *S. diffusa* | NTM C14464 | N. Australia | FJ621400 | KU230377 |
| *S. finitima* | RMNH Coel. 41331 | Palau | KF915731 | KF955204 |
| *S. flaccida* | RMNH Coel. 38731 | Palau | FJ621408 | NA |
| *S. flexibilis* | RMNH Coel. 38378 | Indonesia, Ambon | FJ621409 | NA |
| *S. flexibilis* | RMNH Coel. 41315 | Palau | KF915737 | KF955210 |
| *S. foliata* | RMNH Coel. 38732 | Palau | FJ621410 | NA |
| *S. gaweli* | UF3498 | Guam | FJ621417 | NA |
| *S. gaweli* | UF3181 | Guam | FJ621418 | NA |
| *S. gaweli* | RMNH Coel. 41338 | Palau | KF915741 | KF955214 |
| *S. gibberosa* | ZMTAU Co 33611 | Taiwan, Penghu | JX991171 | JX991260 |
| *S. grandilobata* | NTM C13796 | Palau | FJ621420 | NA |
| *S. grandilobata* | RMNH Coel. 41312 | Palau | KF915744 | KF955217 |
| *S. grandilobata* | RMNH Coel. 41313 | Palau | KF915745 | KF955218 |
| *S. gravis* | RMNH Coel. 41328 | Palau | KF915746 | KF955219 |
| *S. heterospiculata* | NTM C13968 | Mauritius | FJ621425 | NA |
| *S. heterospiculata* | NTM C14003 | Vanuatu | FJ621426 | NA |
| *S. heterospiculata* | RMNH Coel. 41320 | Palau | KF915747 | KF955220 |
| *S. humilis* | RMNH Coel. 38737 | Palau | FJ621432 | NA |
| *S. humilis* | RMNH Coel. 41318 | Palau | KF915752 | KF955227 |
| *S. levi* n. sp. | ZMTAU Co 34106 | Red Sea, Israel | FJ621465 | GU355986 |
| *S. levi* n. sp. | ZMTAU Co 34138 | Red Sea, Israel | FJ621466 | GU355986 |
| *S. levi* n. sp. | ZMTAU Co 36585 | Red Sea, Israel | KU230366 | KU230378 |
| *S. levi* n. sp. | ZMTAU Co 36607 | Red Sea, Israel | KU230367 | KU230379 |
| *S. linnei* | NTM C14480 | N. Australia | FJ621440 | KU230380 |
| *S. loyai* | ZMTAU Co 34154 | Red Sea, Israel | FJ621442 | GU355979 |
| *S. luxuriosa* | RMNH Coel. 38742 | Palau | FJ621443 | NA |
| *S. mammifera* | NTM C14198 | Vanuatu | FJ621444 | KU230381 |
| *S. nanolobata* | RMNH Coel. 38441 | Indonesia, Ambon | FJ621451 | NA |
| *S. notanda* | NTM C14555 | N. Australia | FJ621452 | NA |
| *S. numerosa* | NTM C13790 | Palau | FJ621453 | NA |
| *S. ornata* | NTM C14138 | American Samoa | FJ621455 | NA |
| *S. ornata* | ZMTAU Co 34646 | Taiwan, Penghu | JX991173 | JX991263 |
| *S. papula* | NTM C14527 | N. Australia | FJ621456 | KU230382 |
| *S. pavida* | RMNH Coel. 38744 | Palau | FJ621459 | NA |
| *S. peculiaris* | NTM C14092 | Micronesia, Yap | FJ621461 | NA |
| *S. peculiaris* | ZMTAU Co 34643 | Taiwan, Penghu | JX991182 | JX991272 |
| *S. peculiaris* | ZMTAU Co 34707 | Taiwan, Penghu | JX991180 | JX991270 |
| *S. polydactyla* | ZMTAU Co 34140 | Red Sea, Israel | FJ621386 | GU355989 |
| *S. polydactyla* | ZMTAU Co 34142 | Red Sea, Israel | FJ621387 | GU355989 |
| *S. polydactyla* | ZMTAU Co 34150 | Red Sea, Israel | FJ621388 | GU355989 |
| *S. polydactyla* | USNM 1202015 | Red Sea, Saudi Arabia | KC864920 | KC864989 |
| *S. polydactyla* | ZMTAU Co 34181 | Red Sea, Israel | KU230374 | GU355987 |
| *S. procera* | RMNH Coel. 38386 | Indonesia, Ambon | FJ621467 | NA |
| *S. querciformis* | ZMTAU Co 34096 | Red Sea, Israel | FJ621469 | GQ342399 |
| *S. querciformis* | ZMTAU Co 34191 | Red Sea, Israel | FJ621470 | GU355985 |
| *S. rigida* | NTM C14141 | Am. Samoa | FJ621472 | NA |
| *S. scabra* | NTM C14043 | Vanuatu | FJ621476 | NA |
| *S. ?scabra* | RMNH (PBH-C6) | Guam | KU230368 | KU230384 |
| *S. ?scabra* | RMNH (PBH-C10) | Guam | KU230369 | KU230383 |
| *S. siaesensis* | RMNH Coel. 38746 | Palau | FJ621477 | NA |
| *S. siaesensis* | RMNH Coel. 38747 | Palau | FJ621478 | NA |
| *S. sobolifera* | RMNH Coel. 38748 | Palau | FJ621479 | NA |
| *S. sublimis* | RMNH Coel. 41323 | Palau | KF915760 | KF955235 |
| *S. tumulosa* | RMNH Coel. 41325 | Palau | KF915761 | KF955236 |
| *S. tumulosa* | RMNH Coel. 41329 | Palau | KF915762 | KF955237 |
| *S. ultima* | RMNH Coel. 41324 | Palau | KF915763 | KF955238 |
| *S. uniformis* | RMNH Coel. 38753 | Palau | FJ621484 | NA |
| *S. variabilis* | NTM C14134 | American Samoa | FJ621485 | NA |
| *S. variabilis* | NTM C14164 | Papua New Guinea | FJ621486 | NA |
| *S. verruca* | RMNH Coel. 38754 | Palau | FJ621487 | NA |
| *S. verruca* | RMNH Coel. 41340 | Palau | KF915764 | KF955239 |
| *S. verruca* | RMNH Coel. 41341 | Palau | KF915765 | KF955240 |
| *S. vrijmoethi* | NTM C14095 | Micronesia, Yap | FJ621489 | NA |
| *S. woodyensis* | NTM C14557 | N. Australia | FJ621490 | KU230389 |
| *Sinularia* sp. | NTM C14142 | American Samoa | FJ621462 | NA |
| *Sinularia* sp. | NTM C14173 | Papua New Guinea | FJ621463 | NA |
| *Sinularia* sp. | RMNH Coel. 41339 | Palau | KF915753 | KF955228 |
| *Sinularia* sp. | RMNH (PBH-To1) | Guam | KU230370 | KU230385 |
| *Sinularia* sp. | RMNH (PBH-To4) | Guam | KU230371 | KU230386 |
| *Sinularia* sp. | RMNH (PBH-To5) | Guam | KU230372 | KU230387 |
| *Sinularia* sp. | RMNH (PBH-Tr3) | Guam | KU230373 | KU230388 |
